# Mouse TAPBPR shows functional similarity to human TAPBPR in shaping the MHC-I immunopeptidome

**DOI:** 10.3389/fimmu.2026.1756668

**Published:** 2026-04-15

**Authors:** Juliana Bernardi Aggio, Marcel Wacker, William Guo, Arwen F. Altenburg, Ida Hafstrand, Reem Satti, Alison McDowall, Aure Aflalo, Andreas Neerincx, Jack Welland, Alice Abreu Torres, Mikkel-Ole Skjoedt, Karsten Skjødt, Jens Bauer, Juliane S. Walz, Louise H. Boyle

**Affiliations:** 1 Department of Pathology, University of Cambridge, Cambridge, United Kingdom; 2 Department of Peptide-based Immunotherapy, Institute of Immunology, University and University Hospital Tübingen, Tübingen, Germany; 3 Cluster of Excellence iFIT (EXC2180) “Image-Guided and Functionally Instructed Tumor Therapies”, University of Tübingen, Tübingen, Germany; 4 Cambridge Institute for Medical Research, University of Cambridge, Cambridge, United Kingdom; 5 Rigshospitalet-University Hospital Copenhagen, Copenhagen, Denmark; 6 Institute of Immunology and Microbiology, University of Copenhagen, Copenhagen, Denmark; 7 University of Southern Denmark, Odense, Denmark; 8 German Cancer Consortium (DKTK), Partner Site Tübingen, A Partnership Between DKFZ and University Hospital Tübingen, Tübingen, Germany; 9 Clinical Collaboration Unit Translational Immunology, Department of Internal Medicine, University Hospital Tübingen, Tübingen, Germany

**Keywords:** calnexin, H2-Db, H2-Kb, immunopeptidome, MHC-I, mouse TAPBPR, peptide exchange

## Abstract

Human TAPBPR is known to function as a Major Histocompatibility Complex class I (MHC-I) peptide exchange catalyst that shapes the peptide repertoire presented to immune cells. However, investigations characterizing TAPBPR from other species are limited. Here, we characterize mouse TAPBPR, exploring its association partners in mouse cell lines and comparing its function to human TAPBPR. We find that mouse TAPBPR binds MHC-I and calnexin, with a notably sustained interaction with H2-D^b^ compared to H2-K^b^. We reveal that mouse TAPBPR restricts the peptide repertoire presented on H2-D^b^ and H2-K^b^ on MC-38 cells. Intriguingly, mouse TAPBPR presence promotes the selection of peptides with a C-terminal methionine on H2-K^b^. We reveal that in the presence of high-affinity peptides, mouse TAPBPR can promote loading of both H2-D^b^ and H2-K^b^. Furthermore, mouse TAPBPR efficiently loaded a peptide with a C-terminal methionine onto H2-K^b^. Together, our findings suggest that mouse TAPBPR plays an important role in shaping the MHC-I immunopeptidome by functioning as a peptide editor, similar to its human counterpart.

## 1 Introduction

Major Histocompatibility Complex class I (MHC-I) molecules play a vital role in immune recognition by presenting a fraction of a cell’s peptidome on the plasma membrane for monitoring by potent cytotoxic cells such as CD8+ T lymphocytes. Within the endoplasmic reticulum (ER), MHC-I heavy chains initially fold with the help of the membrane-bound chaperone calnexin (1–3). Upon formation of a beta2-microglobulin (β2m)-associated MHC-I heavy chain dimer, calnexin is replaced by its soluble ER luminal counterpart calreticulin for human MHC-I, while mouse MHC-I:β2m dimers can remain associated with calnexin (4–6). The selection of antigenic cargo onto intracellular MHC-I is aided by two related molecular chaperones, tapasin and TAP binding protein-related (TAPBPR). While both tapasin and TAPBPR can perform peptide exchange on MHC-I (7–10) to assist in selecting the peptide repertoire presented, they appear to work in distinct molecular settings. Tapasin is known to function in the peptide-rich environment of the peptide loading complex (PLC) in association with the transporters associated with antigen processing (TAP), calreticulin and ERp57 (11–14). Tapasin promotes peptide loading onto MHC-I, with many MHC-I molecules dependent on tapasin for stable surface expression (15–17). In contrast, human TAPBPR is not an integral component of the PLC and can work in conjunction with UDP-glucose: glycoprotein glucosyltransferase 1 (UGGT1) (18–20). Thus far, human TAPBPR does not appear to be essential for the surface expression of human MHC-I molecules, with TAPBPR playing a fine-tuning role in the immunopeptidome, restricting the peptide repertoire expressed (9, 19, 21, 22).

Our understanding of the mechanism of MHC-I peptide editing by tapasin and TAPBPR has advanced significantly in recent years through several insightful structural studies (23–27). These studies have confirmed that both chaperones bind to a similar site on MHC-I and have revealed that the key step in peptide editing is the widening of the MHC-I peptide binding groove. An editing loop has been identified on both tapasin and TAPBPR, which sits adjacent to the MHC-I F pocket, which is involved in binding to the C-terminus of peptide cargo. While different mechanisms of action for these editing loops have been proposed (21, 26, 28–31), this region is clearly contributing to the peptide editing process.

Most of our understanding of TAPBPR thus far stems from molecular, biochemical, structural, and biophysical studies of human TAPBPR. Surprisingly, very little characterization of TAPBPR from other species has been performed at a protein or functional level. The most substantial study to date compared the ability of human, chicken and mouse TAPBPR to interact with MHC-I molecules from humans, human leukocyte antigens (HLA) (32). While human TAPBPR has been shown to preferentially interact with HLA-A molecules of the A2 and A24 supertypes (33), Sun et al. revealed that chicken TAPBPR has broader allele specificity to human MHC-I (32). In contrast, mouse TAPBPR formed complexes only with HLA-A*02:01 and exhibited weak interactions with all other HLA allotypes tested (32). Thus, while we know that some mouse MHC-I molecules (e.g. H2-D^b^, L^d^ and H2-D^d^) can bind to and undergo peptide exchange by human TAPBPR (10, 23, 26), very little is known regarding the role of mouse TAPBPR in MHC-I antigen processing and presentation.

Unexpectedly, an alternative function for TAPBPR as a negative regulator of T cells in mice has also been proposed (34). This suggestion was based on the relatedness of the IgV and IgC domains of TAPBPR to B7 family members, the apparent detection of low levels of mouse TAPBPR on the surface of immune cells and some cancer cells, and administration of a human TAPBPR-IgG fusion protein reducing T cell activation and function (34).

Given the lack of thorough investigation into TAPBPR from any non-human species in an autologous system, we set out to investigate the function of mouse TAPBPR in cell lines derived from C57BL/6 mice, employing similar methodologies we previously used for human TAPBPR (9, 18, 19, 35). Here, mass spectrometry analysis of mouse TAPBPR immunoprecipitates confirmed its interaction with MHC-I. Immunopeptidomic analysis reveals that mouse TAPBPR shapes the peptide repertoire presented on both H2-D^b^ and H2-K^b^. This work suggests that mouse TAPBPR may function as an MHC-I peptide editor, playing a key role in antigen processing and presentation, as previously described for human TAPBPR. With the prior suggestion for a dominant role for mouse TAPBPR on the cell surface (34), we also explored whether mouse TAPBPR expressed at physiological levels was detectable at the plasma membrane, but we were unable to detect significant amounts at this site. However, like its human counterpart (35), the overexpression of mouse TAPBPR in cell lines using lentiviral transduction can cause its leakage to the plasma membrane and can be utilized as an assay system to explore the ability of mouse TAPBPR to promote peptide loading onto both H2-D^b^ and H2-K^b^.

## 2 Material and methods

### 2.1 Cells

Murine colon adenocarcinoma MC-38 (Kerfast, Newark, CA 94560, USA), mouse melanoma B16-F10, Lewis lung carcinoma LL/2 and mouse stromal fibroblast MEF-BL/6-1 (ATCC SCRC-1008) cells were maintained in Dulbecco’s Modified Eagle’s medium (DMEM)(CAT: 41966052, Gibco™, Thermo Fisher Scientific, Paisley, Renfrewshire, UK), supplemented with 10% fetal bovine serum (FBS)(CAT: 10500064, Gibco™) and 100 units/mL penicillin-streptomycin (CAT: 15140122, Gibco™) at 37 °C, 5% CO_2_, and humid atmosphere. Human-derived cell lines HeLaM and HEK 293T (both gifts from Paul Lehner, University of Cambridge, UK) were cultured under the same conditions. For MC-38 cells, DMEM media was additionally supplemented with 0.01 M HEPES (CAT:15630056, Gibco™) and 1X MEM non-essential amino acids solution (CAT: 11140035, Gibco™). All cells were regularly tested for the absence of mycoplasma using the MycoAlert kit (Lonza; CAT: LT07-318, Lonza Group AG Basel, Switzerland). Unless indicated otherwise., cells were treated for 48 hours with 200 U/mL of murine IFN-gamma (IFNγ)(CAT:315-05, PeproTech, Thermo Fisher UK Limited, Altrincham, Cheshire, UK) to upregulate peptide presentation pathway components.

### 2.2 Generation of knockout cell lines

Mouse TAPBPR (*Tapbpl* NCBI gene ID 213233) was knocked out in MC-38 cells using CRISPR-Cas9, with the transient transfection system as has been described before (9, 36), using g4:rev AAACCGGGGCTTTTGCCAGCAGTGC. Single-cell clones were produced, and a single MC-38 TAPBPR knockout clone was used in further applications. H2-K^b^ (*H2-K1* gene ID 14972), H2-D^b^ (*H2-D1* gene ID 1964) and β2m (beta-2 microglobulin gene ID 12010) were targeted to generate MHC-I-deficient cell lines in B16-F10, MEF-BL/6-1, MC-38 and the MC-38-TAPBPR knock-out cells using CRISPR-Cas9 ribonucleoprotein (RNP) delivered by nucleofection. Alt-R CRISPR-Cas9 crRNAs (Integrated DNA Technologies UK Ltd. (IDT), London, UK) were designed by IDT’s Custom Alt-R CRISPR-Cas9 guide RNA tool to target H2-K^b^ (F’ GTACATGGAAGTCGGCTACG), H2-D^b^ (F’ GAGCCCCGGTACATCTCTGT) or β2m (F’ CCGAGCCCAAGACCGTCTAC). As a control, cells underwent RNP-nucleofection with the negative control RNA guide.

To form the guide RNAs, 0.6 nmol of each crRNA was incubated with 0.6 nmol of Alt-R CRISPR-Cas9 tracrRNA (IDT) for 5 minutes at 95 °C. The guide RNA was incubated for 20 minutes at room temperature with 85 µg of Alt-R S.p. Cas9 Nuclease V3 (CAT:1081058, IDT) in a final volume of 25 μL per reaction, in phosphate-buffered saline (PBS)(Gibco™). Each RNP was added to 2x10^6^ target cells (washed three times in advance) in combination with 1 nmol of Alt-R Cas9 Electroporation Enhancer (CAT: 1075915, IDT), and 100 μL of Cell Line Nucleofector Kit V (Lonza) for MC-38 and B16-F10 cells, or Kit L for MEF-BL/6–1 cells on a 1:4.5 ratio of Supplement and Nucleofector Solution (Lonza). RNP-nucleofection was performed in the Nucleofector II/2b device (Lonza), running program D-032 (MC-38 cells), P-020 (B16-F10 cells), or A-023 (MEF-BL/6-1). After 7 days, cells knocked out for H2-K^b^, H2-D^b^ or β2m were sorted by flow cytometry for low MHC-I surface expression following IFNγ treatment. Those cells rested for an extra 7 days to return to their baseline state. In B16-F10 and MEF-BL/6–1 cell derivatives, exon 2 of TAPBPR (F’ CATCATCTTAGACTGCTTCT) was subsequently targeted for deletion using CRISPR-Cas9 RNP nucleofection, as indicated above. The final TAPBPR knockout cells in B16-F10 and MEF-BL/6–1 cell derivatives were produced by testing single cell clones for loss of TAPBPR by flow cytometry, then combining 25 TAPBPR-negative single cells to make a bulk TAPBPR knockout population in each of the cell lines.

### 2.3 Over-expression of full-length mouse TAPBPR in cell lines

Mouse TAPBPR was cloned from liver cDNA from a C57BL/6 mouse into the lentiviral vector pHRSIN-C56W-UbEM, as used previously for human TAPBPR (18), then subsequently subcloned into pHRSIN-SFFV-MCS(+)-WPRE-PGK-Puro plasmid (a kind gift from Dick Van Den Boomen and Paul Lehner, University of Cambridge, UK). For overexpression of mouse TAPBPR, the mTAPBPR-pHRSIN-C56W-UbEM plasmid was used to transduce HeLaM and LL/2 cells, while the mTAPBPR-pHRSIN-SFFV-MCS(+)-WPRE-PGK-Puro plasmid was used for transductions in MC-38, B16-F10, and MEF-BL6–1 cells. HEK 293T cells were transfected with 800 ng of the lentiviral plasmid, 500 ng packaging plasmid pCMVΔR8.91, 500 ng envelope plasmid pMD.G as previously described (18) and 4.5 μL FuGENE HD Transfection Reagent (CAT: E2311, Promega UK Ltd., Chilworth, Hampshire, UK) in Opti-MEM (Gibco™, Thermo Fisher Scientific) per reaction. Progeny lentivirus was harvested after 48 and 72 hours, filtered (0.45 µm) and transduced on 2x10^5^ target cells in the presence of 10 µg/mL Polybrene (Sigma Aldrich, Merck Life Science UK Ltd., Gillingham, Dorset). When mTAPBPR-pHRSIN-SFFV-MCS(+)-WPRE-PGK-Puro was used, puromycin-resistant cells were selected. Surface and intracellular TAPBPR overexpression (OE) levels were confirmed by flow cytometry. If indicated, some mouse TAPBPR OE cells were sorted to express higher surface TAPBPR.

### 2.4 Expression and purification of recombinant soluble mouse and human TAPBPR

The ectodomain of murine TAPBPR was tagged at the C-terminal with a 6xHis tag and cloned into the PB-T-PAF vector (**Supplementary Figure 1A**). Recombinant soluble mouse or human TAPBPR proteins were then expressed in HEK 293T cells using the PiggyBac expression system and purified as previously described (35). HEK 293T cells expressing His-tagged soluble murine or human TAPBPR were maintained in selection medium (DMEM/10% FBS/1% Pen-Strep, supplemented with 3 μg/ml puromycin and 700 μg/ml geneticin). Protein expression was induced for 7 days with 2 μg/ml doxycycline in DMEM/5% FBS, after which the cell medium was harvested and filtered to remove debris. The soluble TAPBPR proteins were purified using Ni-NTA affinity chromatography (HisTrap™ excel, Cytiva) on an Äkta Start system (Cytiva). After elution in 250 mM Imidazole in PBS/400 mM NaCl, the proteins were concentrated using a Vivaspin 20–30000 MWCO PES concentrator (Sartorius) and further purified using a HiLoadR 16/600 SuperdexTM 75 pg size-exclusion column (Cytiva).

### 2.5 Generation of mouse TAPBPR-specific monoclonal antibodies

Monoclonal antibodies were raised in rats against recombinantly expressed mouse TAPBPR using standard methods as described in Skjoedt et al. (37).

### 2.6 Protein deglycosylation

N-linked oligosaccharides from 20 µg of recombinant soluble TAPBPR were removed by PNGase F (CAT: P0704S, New England Biolabs (UK) Ltd., Hitchin, Hertfordshire, UK) treatment in denaturing reaction conditions, according to the manufacturer’s instructions. The deglycosylation mobility shift was assessed in Bis-Tris gel stained with Coomassie.

### 2.7 Protein melting profile

The protein melt curve was determined by Differential Scanning Fluorimetry (DSF) in reactions of 250 mg/mL of mouse or human soluble TAPBPR (sTAPBPR) protein with 2.5 µl of 5X SYPRO Orange Protein Gel Stain (CAT: S6650, Invitrogen, Thermo Fisher Scientific) made up to 25 µl. The assay was performed in a V-shaped solid white 96-well qPCR plate (Bio-Rad Laboratories Ltd., Watford, Hertfordshire, UK). Fluorescence on the FAM channel was monitored on a CFX96TM Real-Time Thermal Cycler (Bio-Rad Laboratories Ltd) between 25 and 95 °C in continuous ramp mode with a 0.5 °C/s increase in temperature. Fluorescence values were normalized to a percentage. V50 melting temperatures were calculated using GraphPad Prism 5 via fitting to a non-linear Boltzmann Sigmoidal distribution.

### 2.8 Identification of TAPBPR and MHC-I interaction partners

Variant MC-38 and B16-F10 cells were seeded at 3.5x10^5^ and 5x10^5^, respectively, per 100 x 20 mm dishes with four dishes used per cell variant for immunoprecipitation (IP). After 48 hours of IFNγ treatment, cells were harvested using 0.05% trypsin-EDTA, washed twice with PBS and snap-frozen in dry ice.

#### 2.8.1 Immunoprecipitation

Approximately 8x10^6^ cells were lysed in 5 mL of tris buffer saline (TBS) (2 mM Tris-HCl pH 7.4, 150 M NaCl, and 2.5 M CaCl_2_) supplemented with 1% digitonin (High purity, CAT: 300410, Merck Life Science UK Ltd., Gillingham, Dorset, UK), cOmplete mini EDTA-free protease inhibitor tablet (CAT: 04693159001, Roche Diagnostics Ltd., Burgess Hill, Sussex, UK), and 10 mM N-ethylmaleimide (CAT: E3876, Merck), and rocked for 2 hours at 4 °C. Debris was removed by centrifugation at 13,000 x *g* for 20 min at 4 °C. Following removal of 40 µL whole cell lysate as a loading control, the remaining lysate was pre-cleared on Protein G Sepharose FF Resin (CAT: Fastback-PG-5, Clinisciences Ltd., Slough, UK) and IgG Sepharose 6 Fast Flow (CAT: 17096901, Cytiva Global Life Sciences Solutions Operations Ltd., Little Chalfont, Buckinghamshire, UK) for 30 minutes at 4 °C. For immunoprecipitation, Protein G beads were preconjugated with either 5 µg of rat α-mTAPBPR (AnDi38, in house), 5 µg mouse α-H2-K^b^ (Y-3 clone, CAT: Y100-1MG, Thermo Fisher Scientific) or 10 μg mouse α-H2-D^b^ (B22-249.R1 clone, CAT: MA5-17992, Thermo Fisher Scientific) per sample for 30 minutes at 4 °C. The bead:antibody-conjugates were subsequently incubated with the pre-cleared lysates for 2 hours at 4 °C. Following binding, the complexes were washed four times with 0.1% digitonin in TBS.

#### 2.8.2 LC-MS/MS

TAPBPR immunoprecipitates for LC-MS/MS were generated as described above, in triplicate, in a sterile environment, with the last wash in TBS only. The beads were subsequently washed four times with 50 mM ammonium bicarbonate, five times the volume of the beads, by gentle vortexing and spinning at 1500 g for 30 seconds. Following the discard of the final wash, the beads were reduced by incubation in 5 mM Dithiothreitol for 30 minutes at 37 °C, then alkylated by incubation in 15 mM iodoacetamide for 30 minutes at room temperature in the dark. Proteins were digested in 1 μg Sequencing Grade Modified Trypsin (CAT: V5111, Promega) for 16 hours at 37 °C. After digestion, the samples were spun gently, and the digest was collected into a new tube, ensuring it was free of beads. The samples were then loaded onto an autosampler for automated LC-MS/MS analysis.

LC-MS/MS were performed using a Dionex Ultimate 3000 RSLC nanoUPLC (Thermo Fisher Scientific) system and a Q Exactive Orbitrap mass spectrometer (Thermo Fisher Scientific). Separation of peptides was performed by reverse-phase chromatography at a flow rate of 300 nL/min and a Thermo Scientific reverse-phase nano Easy-spray column (Thermo Scientific PepMap C18, 2mm particle size, 100A pore size, 75 mm i.d. x 50cm length). Peptides were loaded onto a pre-column (Thermo Scientific PepMap 100 C18, 5mm particle size, 100A pore size, 300 mm i.d. x 5mm length) from the Ultimate 3000 autosampler with 0.1% formic acid for 3 minutes at a flow rate of 15 mL/min. After this period, the column valve was switched to allow elution of peptides from the pre-column onto the analytical column. Solvent A was water + 0.1% formic acid and solvent B was 80% acetonitrile, 20% water + 0.1% formic acid. The linear gradient employed was 2-40% B in 90 minutes. Further wash and equilibration steps gave a total run time of 120 minutes.

The LC eluant was sprayed into the mass spectrometer by means of an Easy-Spray source (Thermo Fisher Scientific Inc.). All m/z values of eluting ions were measured in an Orbitrap mass analyzer, set at a resolution of 35000 and were scanned between m/z 380–1500. Data-dependent scans (Top 20) were employed to automatically isolate and generate fragment ions by higher energy collisional dissociation (HCD, NCE:26%) in the HCD collision cell and measurement of the resulting fragment ions was performed in the Orbitrap analyzer, set at a resolution of 17500. Singly charged ions and ions with unassigned charge states were excluded from being selected for MS/MS and a dynamic exclusion window of 40 seconds was employed.

#### 2.8.3 Protein database searching

Following LC-MS/MS, the data were processed using Protein Discoverer (version 2.5, ThermoFisher). Briefly, MS/MS data were converted to mgf files and submitted to the Mascot search algorithm (Matrix Science) and searched against a common contaminants database and the UniProt mouse database. Variable modifications of oxidation (M) and deamidation (NQ) and a fixed modification of carbamidomethyl (C) were applied. Peak areas for each identified peptide were generated and combined to give protein abundance values. The peptide and fragment mass tolerances were set to 20 ppm and 0.1 Da, respectively.

Scaffold_5.1.1 Proteome Software was used to validate MS/MS based peptide and protein identifications. Peptide identifications were accepted if they could be established at greater than 95.0% probability by the Scaffold Local FDR algorithm. Protein identifications were accepted if they could be established at greater than 99.0% probability and contained at least 2 identified peptides. Protein probabilities were assigned by the Protein Prophet algorithm (38).

Data were further processed with in-house Python scripts using Numpy (39), Pandas (40), SciPy.stats (41), matplotlib (42), and seaborn (43) packages. MHC-I-derived peptides were compared to the sequence H2-K^b^, H2-D^b^ or H2-M3. Peptide sequences uniquely identified within these specific MHC-I sequences were assigned to the corresponding allotype. Peptide sequences that were corresponded to more than one MHC-I sequence, i.e. peptides derived from conserved regions, were combined under ‘MHC-I’ as the specific allotype that the peptide derived from could not be established. Subsequently, the dataset was filtered to exclude peptides that matched the reverse database, and proteins that were detected in a single repeat, detected by <4 peptides (except for MHC-I), or were keratin, immunoglobulin or trypsin. To compare TAPBPR KO and OE cells, missing signal intensity values were imputed using the half-minimum of the relevant sample, allowing for calculation of fold-change per repeat. P-value was calculated using an independent t-test (one-tailed) with Benjamini-Hochberg correction.

#### 2.8.4 Immunoblotting

Lysates and immunoprecipitates were produced as described above using digitonin. As an antibody-only control, protein G and antibody-preconjugated beads, i.e., with no lysate, were used. Where specifically indicated, total cell lysates were also produced in TBS supplemented with 1% Triton-X (CAT: 28817.295, VWR International Ltd., Lutterworth, Leicestershire, UK) with the addition of a protease inhibitor tablet. Prior to separation using gel electrophoresis, samples were mixed with Laemmli sample buffer (50 nM Tris-HCl, pH 6.8, 2% SDS, 10% glycerol, and 0.1% bromophenol blue [w/v]) supplemented with 100 nM 2-Mercaptoethanol if reducing conditions were required, and heated at 95°C for 5min.

Following protein separation on NuPAGE 10% Bis-Tris Gels or NuPAGE 4-12% Bis-Tris Gels (CAT: NP0301BOX and NP0322BOX, Invitrogen, Thermo Fisher Scientific, Altrincham, Cheshire, UK), the gels were equilibrated in Transfer buffer (2.5 mM Tris, 19.2 mM glycine, and 20% methanol [w/v]) and transferred onto Amersham Protran Premium 0.2um NC Nitrocellulose Blotting Membrane (CAT: 10600004, Global Life Sciences Solutions Operations Ltd., Little Chalfont, Buckinghamshire, UK) in the Trans-Blot Turbo Transfer System (Bio-Rad). Membranes were blocked for 30 minutes at room temperature in 5% (wt/vol) dried milk and 1% (vol/vol) Tween 20 in PBS and incubated for 24 hours at 4 °C with primary antibodies in the same buffer: rat α-mTAPBPR (AnDi25, *in house*), rabbit α-MHC-I (HLA class I ABC, CAT: 15240-1-AP, Proteintech), rabbit α-β2m (CAT: A0072, Dako UK Ltd, ELY, Cambridgeshire, UK), rabbit α-calnexin (CAT: ADI-SPA-860, Enzo Life Sciences UK Ltd., Exeter, Devon, UK), rabbit α-tapasin (CAT: ab288565, Abcam Ltd., Cambridge, Cambridgeshire, UK), rabbit α-TAP2 (CAT: A13547, Antibodies.com, Cambridge, UK), rabbit α-GAPDH (CAT: PA1-897, Thermo Fisher Scientific), rabbit α-H2-K^b^ (MHC class I, CAT: ab281902, Abcam Ltd), and rat α-H2-D^b^ (MHC class I, CAT: sc-59199, Santa Cruz Biotechnology distributed by Insight Biotechnology Ltd., Wembley, UK). After three washes with 1% Tween 20 in PBS, the membranes were incubated with conjugated species-specific secondary antibodies in 1% Tween 20 in PBS, added with 5% milk for 1 hour at room temperature: IRDye 800CW goat α-rat IgG (CAT: 926-32219, LI-COR Biosciences UK Ltd., Cambridge, Cambridgeshire, UK) and IRDye 800CW goat α-rabbit IgG (CAT: 926-32211, LI-COR Biosciences UK Ltd.). Membranes were washed three times with 1% Tween 20 in PBS and once with PBS, and then visualized using a Typhoon 5 Imager (CAT: 29187191, Cytiva Global Life Sciences Solutions Operations Ltd., Little Chalfont, Buckinghamshire, UK). In blotting experiments using antibodies against mTAPBPR, H2-K^b^, and H2-D^b^, non-reducing conditions were used.

### 2.9 MHC-I immunopeptidomics

Knockout variants of MC-38 and B16-F10 were seeded in T175 flasks and stimulated with IFNγ. After 48 hours, cells were dislodged (MC-38) or harvested using 0.5 mM EDTA in PBS (B16-F10), washed three times in PBS, pelleted and snap-frozen. Pellets were stored at -70 °C before peptide isolation.

#### 2.9.1 Isolation of H2-D^b^ and H2-K^b^

H2 molecules were immunopurified by immunoaffinity chromatography from 5x10^8^ cells using the H2-D^b^ and H2-K^b^ specific monoclonal antibodies, B22.249 and Y-3, respectively. A CHAPS-based lysis buffer (1.2% [w/v] in PBS, supplemented with Protease inhibitor cocktail containing EDTA [Roche]) was used to lyse MC-38 and B16-F10 cells. The immunoprecipitation was performed using Econo Column Chromatography Columns (0.5cm × 5cm or 5cm × 5cm, Bio-Rad). Subsequently, peptides were isolated by acidic elution with 0.2% (v/v) trifluoroacetic acid (TFA), size-exclusion filtration with an Amicon Ultra 0.5 centrifugal filter unit (Merck Millipore), followed by a desalting step using a ZipTip C18 pipette tip (Merck-Millipore).

#### 2.9.2 Analysis of peptides by mass spectrometry

Isolated peptides were analyzed from five technical replicates. Reversed-phase liquid chromatography (nanoUHPLC, UltiMate 3000 RSLCnano, Thermo Fisher Scientific), was carried out applying a gradient ranging from 2.4 to 32.0% of ACN for 90 min for sample separation followed by an on-line coupled Orbitrap Fusion Lumos mass spectrometer (Thermo Fisher Scientific), using data dependent acquisition and a collision induced dissociation method generating fragment spectra with a mass range limited to 400–650 m/z and positive charge states 2-3.

#### 2.9.3 Database search and peptide filtering

Spectra were annotated by database search of the mouse proteome without isoforms (Swiss-Prot, v150629) using the Sequest HT search engine allowing oxidized methionine as dynamic modification. Peptides were filtered to allow a length of 8–12 amino acids and the false discovery rate was set to 5%. H2 binders were identified for each allotype as having either a percentile rank of ≤2% using NetMHCpan 4.1 (44).

#### 2.9.4 Label free quantitation

The peptide amount for all samples was normalized prior to mass spectrometry analysis by cell count. For each pair of conditions analyzed, peptide lists from the five replicates of both conditions were merged and filtered for HLA binders as above. Peptide spectrum matches (PSM), corresponding scores, and intensities (area under the curve of the extracted ion chromatogram of precursor ions) for each replicate were extracted from the unfiltered data and included in the peptide list. For each peptide and each condition, mean area under the curve across all LFQ-MS runs was calculated, and used to determine the fold change of mean area under the curve in condition A versus condition B. Peptides not detected in at least two technical replicates were excluded from Volcano plots. The fold change of peptides detected only in one condition was calculated by replacing zero values with the median of the five least intensities, representing the limit of detection specific to each sample. Further, a normalization step computing PSM intensities in proportion to the total intensity of precursor ions in technical replicates was included. Statistical significance was calculated using a two-tailed t test with correction for multiple comparisons using the Benjamini-Hochberg method.

#### 2.9.5 Bioinformatic analysis

Peptide lists of predicted binders were further processed with original Python 3.7 code using NumPy (39), pandas (40), SciPy.stats (41), matplotlib (42), seaborn (43), and logomaker (45) packages. For semi-quantitative analysis, fold-change and corresponding statistical significance (two-tailed t test with Benjamini-Hochberg correction for multiple comparison) were calculated and the data was visualized in a volcano plot. Enrichment was set at an adjusted p-value of < 0.01 for any up- or down-modulated peptides (log2(fold change)>2 or <-2). NetMHCpan-4.1 (44) was used to predict affinity of peptides per MHC-I allotype. To compare the predicted affinity of the peptide repertoire subsets, a Shapiro-Wilk test was used to determine that the samples were not normally distributed and a Mann-Whitney U test was performed.

### 2.10 Flow cytometry

MC-38 cell line variants were seeded at 3.5x10^5^ cells per 100 x 20 mm dish, while B16-F10 and MEF-BL/6–1 were seeded at 5x10^5^ cells per dish. After 48 hours, cells were harvested using 0.05% trypsin-EDTA (Gibco), washed in FACS buffer (2% FBS [w/v] and 0.5 mM EDTA in PBS) and split in U bottom 96-well plates.

#### 2.10.1 Cell characterization and cell sorting

For intracellular staining, cells were fixed and permeabilized using BD Cytofix/Cytoperm kit (CAT 554715, BD Biosciences). The cells were incubated with antibodies for 30 minutes at 4 °C: rat α-mTAPBPR (AnDi3, in house), mouse α-H2-K^b^ (AF6-88.5, CAT: 116505 or 116517, Biolegend UK Ltd., London, UK), mouse α-H2-D^b^ (KH95, CAT: 111507 or 111513, BioLegend UK Ltd.), rabbit α-β2m (polyclonal, CAT: A0072, Dako), or mouse α-human TAPBPR (PeTe4, in house) (9). After two rounds of washing with FACS buffer (surface staining) or Perm/Wash buffer (intracellular staining), if necessary, a conjugated secondary antibody was added for 30 minutes at 4 °C: goat α-rat IgG (CAT: A-21247, Thermo Fisher Scientific), goat α-rabbit IgG (CAT: A-21244, Thermo Fisher Scientific), or goat α-mouse IgG (CAT: A-21235, Thermo Fisher Scientific). Cells were washed twice and analyzed on a CytoFLEX S Flow Cytometer (Beckman Coulter). Unstained, isotype, or secondary antibody-only controls were included as required. Unless specified, gates were set on total singlet cells (based on morphology).

For cell sorting, 5x10^6^ target cells were harvested and surface-stained (if necessary) in tubes as described above with desired populations isolated using an ARIA III (BD) machine.

#### 2.10.2 Soluble TAPBPR binding assays

To measure the binding of recombinant TAPBPR to surface MHC-I molecules, cells were incubated with 1–1000 nM of recombinant mouse or human TAPBPR in Opti-MEM for 30 minutes at 37 °C. Cells were washed twice with FACS buffer and incubated with rat α-mouse TAPBPR (AnDi3, in-house) or mouse α-human TAPBPR (PeTe4, in-house) antibodies for 30 minutes at 4 °C. If necessary, followed by two rounds of washing with FACS buffer, cells were incubated with species-specific conjugated secondary antibodies for 30 minutes at 4 °C, washed twice, and analyzed by flow cytometry as above.

#### 2.10.3 Plasma-membrane MHC-I peptide binding assays

Fluorescent dye 5-TAMRA (5-carboxytetramethylrhodamine)-labelled derivatives of the following peptides were synthesized (Peptide Synthetics) with the 5-TAMRA position indicated in the peptide sequence by an asterisk (*): SIINFEK*L (derivative of SIINFEKL from Ovalbimun^258-256^, IEDB epitope 58560); SIIRFEK*L (derived from mutant of SIINFEKL, IEDB epitope 143749); SIINFEK*M (mutated version of SIINFEK*L to test alteration of C-terminal L to M); KAVK*NFATC (derivative of KAVYNFATC from LCMV glycoprotein polyprotein(GP)^33-41^, IEDB epitope 30001); KAVK*LFATC (mutated version of KAVK*NFATC used to test alteration of the N at position 5 to L); and FAPGNYPK*L (Derivative of FAPGNYPAL from Sendai virus nucleoprotein^324-332,^IEDB epitope 15248). For all peptides, the TAMRA label was introduced at a position that should not impinge binding to MHC-I. Peptides were prepared according to the manufacturer’s instructions.

The ability of peptides to bind to plasma membrane MHC-I molecules on cells was assessed both in the absence or presence of pretreatment with soluble TAPBPR for 30 min at 37 °C. Following washing to remove any unbound recombinant TAPBPR, cells were incubated with 0–1000 nM of the individual TAMRA-labelled peptides in Opti-MEM for 30 minutes at 37 °C as indicated. To measure TAPBPR-mediated loading peptides on the surface of cells overexpressing TAPBPR, cells were incubated -/+ fluorescent peptides, followed by staining for plasma-membrane expressed TAPBPR, as described above. Following washing three times in FACS buffer, fluorescence was measured using flow cytometry. TAMRA fluorescence was measured inside the total singlet cell population (in controls and cells without OE TAPBPR), or into surface TAPBPR positive populations gated based on similar MFI values in cells OE TAPBPR.

#### 2.10.4 Statistics on flow cytometry data

Flow cytometry data were analyzed using FlowJo V10 (BD Biosciences) with graphs generated using GraphPad Prism 8 (GraphPad Software, Inc.). Unpaired t-test or ordinary one-way ANOVA with Tukey’s multiple comparison test was used where appropriate to compare the average of groups (p-values: (*) p ≤ 0.05, (**) p ≤ 0.01, (***) p ≤ 0.001, (****) p ≤ 0.0001).

## 3 Results

### 3.1 Key MHC-I binding sites on human TAPBPR are conserved on mouse TAPBPR

**Figure 1:**
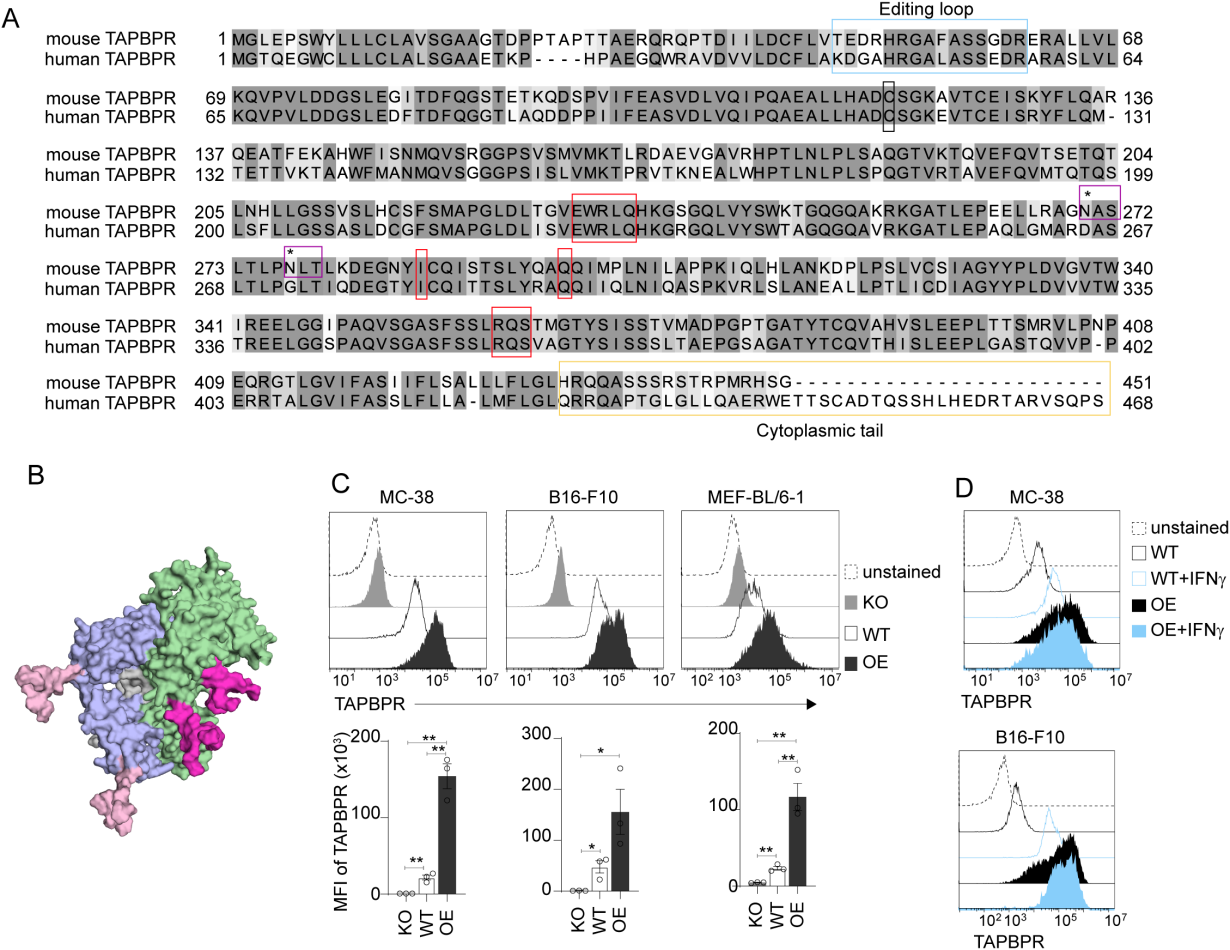
Feature of mouse TAPBPR protein. **(A)** ClustalWS alignment comparison between mouse TAPBPR (mouse TAPBPR, UniProt Q8VD31) and human TAPBPR (UniProt Q9BX59) proteins. Blosum62 scoring system was generated by Jalview 2.11.4.0 software. Boxes highlight the peptide editing loop (blue), MHC-I binding sites characterized as TN5, TN6, TC2 and TC3 (red), the free cysteine residue (black), two predicted N-linked glycosylation sites in mouse TAPBPR (purple, with the asparagine indicated by an asterisk) and the cytoplasmic tail regions (yellow). The endogenous mouse TAPBPR sequence in MC-38, B16-F10, and MEF-BL/6–1 cells was confirmed as equivalent to the UniProt reference. **(B)** Predicted AlphaFold2 structure (46) of mouse TAPBPR (green) bound to H2-D^b^ (blue) with N-linked glycosylations (pink) modelled using GLYCAM (https://glycam.org). For H2-D^b^, only two of the three glycans are visible in the image, with N86 obscured by the orientation depicted. **(C)** Representative histograms and bar graphs showing mean fluorescence intensity (MFI) of intracellular TAPBPR expression, detected using AnDi3 antibody, on IFNγ-treated wildtype (WT) MC-38, B16-F10, and MEF-BL/6–1 cells compared to TAPBPR knockout (KO) and mouse TAPBPR overexpressed (OE) equivalents, which serve as negative and positive controls, respectively. Error bars show MFI -/+ standard error of mean (SEM) from three independent experiments. *p ≤ 0.05, **p ≤ 0.01 using unpaired t-test. **(D)** Histograms showing IFNγ inducibility of intracellular TAPBPR expression in WT MC-38 and B16-F10 cells and in cells transduced to overexpress (OE) mouse TAPBPR.

To begin to explore the properties of mouse TAPBPR, we compared the protein sequences of human and mouse TAPBPR (**Figure 1A**). The two orthologs share 68.6% sequence identity, with the key sites identified on human TAPBPR as being involved in binding to MHC-I conserved in mouse TAPBPR (47). While the TAPBPR editing loop region is also present on mouse TAPBPR, interestingly, the leucine residue in human TAPBPR, suggested to be involved in dissociating peptide from HLA-A molecules (21), is a phenylalanine in mouse TAPBPR (**Figure 1A**). The free cysteine residue at position 94 of mature human TAPBPR, implicated in interacting with UGGT1, is also conserved in mouse TAPBPR (19) (**Figure 1A**). There are some notable differences between the two orthologs. First, mouse TAPBPR has a considerably shorter cytoplasmic tail than human TAPBPR. Second, while human TAPBPR does not contain any N-linked glycosylation sites, mouse TAPBPR has two predicted N-linked glycosylation sites (NXT/NXS motifs) in its lumenal region (**Figure 1A**), which are predicted to be positioned on an exposed surface distal to the MHC-I-binding site (**Figure 1B**). PNGase F treatment of soluble recombinant TAPBPR proteins confirmed that mouse TAPBPR was glycosylated while human TAPBPR was not (**Supplementary Figure 1B**). Thus, variations between orthologs may alter certain properties of TAPBPR.

### 3.2 TAPBPR is expressed in model mouse cell lines

To explore the function of mouse TAPBPR, we generated a panel of rat monoclonal antibodies raised against the lumenal region of this protein. These were tested for their ability to detect mouse TAPBPR in a variety of techniques (**Supplementary Figures 1C–H**). Subsequently, the rat anti-mouse TAPBPR hybridomas, AnDi3 and AnDi38, were selected for detecting mouse TAPBPR in both immunoprecipitation and flow cytometry, while AnDi25 was selected for western blot analysis.

We then tested three model cell lines derived from C57BL/6 mice for their expression of TAPBPR: MC-38, B16-F10, and MEF-BL/6-1. As a negative control, TAPBPR was knocked out in these cells using CRISPR/Cas9, while overexpression of mouse TAPBPR by lentiviral transduction served as a positive control. Intracellular staining followed by flow cytometry demonstrated that TAPBPR was expressed in all three cell lines following IFNγ treatment (**Figure 1C**) as well as the loss of TAPBPR in knockout lines and high expression in cells overexpressing TAPBPR (**Figure 1C**). Like human TAPBPR (18), mouse TAPBPR was IFNγ inducible (**Figure 1D**).

### 3.3 Mouse TAPBPR interacts with MHC-I and calnexin

**Figure 2:**
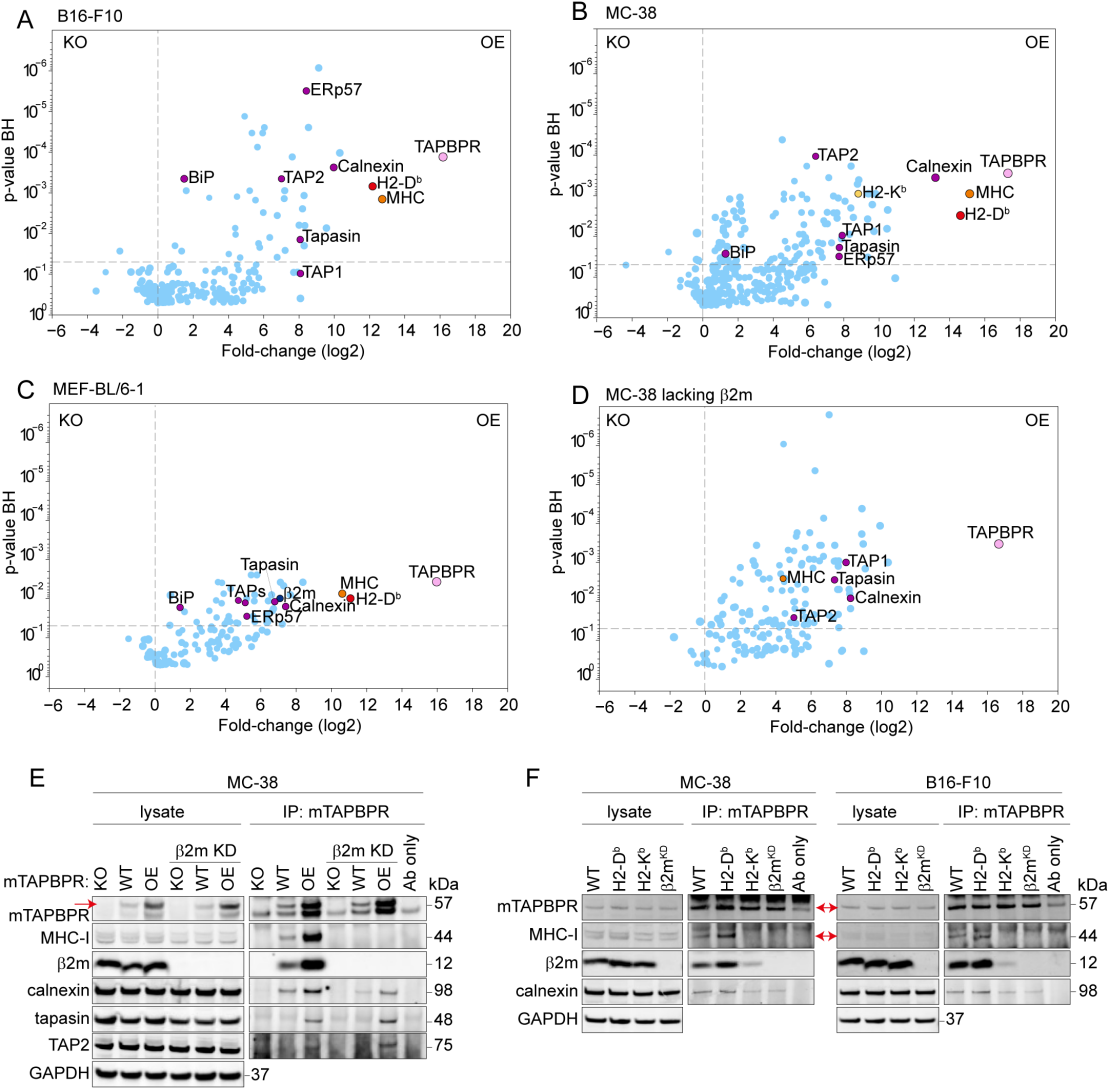
Mouse TAPBPR interaction partners identified in B16-F10, MC-38 and MEF-BL/6–1 cell lines. Mouse TAPBPR was isolated by immunoprecipitation, using Andi38 antibody, from TAPBPR knockout (KO) or mouse TAPBPR overexpressing (OE) from **(A)** B16-F10 cells, **(B)** MC-38 cells, **(C)** MEF-BL/6–1 cells or **(D)** MC-38 cells with β2m knocked out. Scatterplots show all proteins identified via mass spectrometry in the mouse TAPBPR pull-downs in cells overexpressing mouse TAPBPR compared to the equivalent TAPBPR KO cell line. Selected significant interaction partners highlighted are TAPBPR (pink), H2-D^b^ (red), H2-K^b^ (yellow), MHC-I (orange), which covers peptides common to H2 molecules and therefore cannot be assigned to a specific MHC-I molecule, β2m (navy) and known components of the MHC-I antigen presentation pathway (purple). **(E)** Confirmation of mouse TAPBPR binding partners at endogenous TAPBPR levels in MC-38 cells. Immunoblots indicating abundance of mouse TAPBPR (mTAPBPR), MHC-I, β2m, calnexin, tapasin, TAP2, and GAPDH (loading control) in the whole cell lysates and mouse TAPBPR immunoprecipitates (IP: mTAPBPR) from WT MC-38 cells. MC-38 with TAPBPR knocked out (KO) or overexpressing mouse TAPBPR (OE) are included as controls. Cells competent for β2m expression or with β2m knocked down (β2m KD) were compared to assess the importance of the TAPBPR/MHC-I interaction in the observed associations. An antibody-only lane is included to highlight the antibody’s heavy chain used in the immunoprecipitation. N = 1, for tapasin and TAP2 blot. **(F)** Endogenously expressed mouse TAPBPR exhibits a prolonged association with H2-D^b^ compared to H2-K^b^ in both MC-38 and B16 cells. Immunoblots indicating abundance of mTAPBPR, MHC-I, β2m, calnexin, and GAPDH (loading control) in the whole cell lysate and mTAPBPR immunoprecipitated fraction (IP: mTAPBPR) with Andi 38 from MC-38 or B16-F10 WT cells, and variant cell lines expressing H2-D^b^ only (H2-K^b^ knockout), H-2K^b^ only (H2-D^b^ knockout) or lacking efficient expression of both H2-D^d^ and -K^b^ following β2m knock down (KD). Representative of three independent experiments. Note: Arrowheads indicate the positioning of the major TAPBPR and MHC-I bands in the gels, where background bands were present in the immunoprecipitations. The position of TAPBPR relative to the antibody control also varies due to minor changes in running conditions between experiments. Note: WT cells in F were treated with a non-targeting RNA guide in the RNP.

We explored the binding partners of mouse TAPBPR by determining its interactome in MC-38, B16-F10, and MEF-BL/6–1 cell lines. To this end, TAPBPR was isolated by immunoprecipitation from IFNγ-treated cell lines overexpressing mouse TAPBPR and compared to the equivalent immunoprecipitates from their TAPBPR knockout counterparts using LC-MS/MS. Our results demonstrated that mouse TAPBPR binds to MHC-I in all three cell lines (**Figures 2A–C**). Interestingly, while classical MHC-I molecules H2-K^b^ and H2-D^b^ were both identified in MC-38 cells (**Figure 2B**), only H2-D^b^ was confirmed in B16-F10 and MEF-BL/6–1 cells, as no unique peptides for H2-K^b^ were identified (**Figures 2A, C**). UGGT1, which is known to interact with human TAPBPR (19, 20), was not identified as a binding partner for mouse TAPBPR using our pull-down approach. Following MHC-I, calnexin was identified as an abundant association partner for mouse TAPBPR in all three cell lines (**Figures 2A–C**). The association of mouse TAPBPR with calnexin but not UGGT1 was further confirmed using an immunoprecipitation: western blot approach in IFNγ-treated MC-38 cells with endogenous and overexpressed TAPBPR (**Supplementary Figure 2A**).

Unexpectedly, overexpressed mouse TAPBPR was detected in association with components of the peptide loading complex, including tapasin, TAP1, TAP2 and ERp57 in all three cell lines (**Figures 2A–C**). We reasoned that this might be due to either the handover of MHC-I between tapasin and TAPBPR or an artefact of mouse TAPBPR overexpression. To explore the possibility of an indirect association between TAPBPR and components of peptide loading complex via MHC-I, we knocked down β2m in our MC-38 cells (**Supplementary Figures 2B–H**) and determined the mouse TAPBPR interactome in the absence of conformational MHC-I (MHC-I heavy chain/β2m complexes) by immunoprecipitation of TAPBPR followed by LC-MS/MS (**Figure 2D**). As expected, the interaction between TAPBPR and H2-D^b^ and -K^b^ was no longer detectable in the absence of β2m (**Figure 2D**), with only a single peptide, FDSDAENPR, which maps to both classical and nonclassical MHC-I, detectable at low levels. In contrast, overexpressed mouse TAPBPR still interacted with components of the peptide loading complex in the absence of an interaction with MHC-I (**Figure 2D**).

We subsequently employed an immunoprecipitation-western blot approach to investigate TAPBPR interaction partners at more physiological levels of TAPBPR expression following IFNγ treatment of cells with or without β2m expression. This confirmed that in WT MC-38 cells, mouse TAPBPR interacted with the MHC-I heavy chain and β2m (**Figure 2E**) and that the interaction of mouse TAPBPR with MHC-I was dependent on β2m (**Figure 2E**). While the interaction of mouse TAPBPR with calnexin appeared to be reduced in the absence of the TAPBPR: MHC-I association, an interaction between TAPBPR and calnexin was still detectable in β2m-deficient cells (**Figure 2E**; **Supplementary Figure 2I**). This suggests that calnexin can interact directly with mouse TAPBPR and with MHC-I molecules bound to TAPBPR. Although the interaction between overexpressed TAPBPR and TAP and tapasin was confirmed using the immunoprecipitation-western blot approach, we were unable to detect this interaction when TAPBPR was expressed at physiological levels (**Figure 2E**).

### 3.4 TAPBPR exhibits increased binding to H2-D^b^ compared to H2-K^b^ in both MC-38 and B16 cells

The observed binding of mouse TAPBPR for H2-D^b^ over H2-K^b^ (**Figures 2A–C**) suggested a potential binding preference for specific mouse MHC-I allotypes. To explore this further, we knocked out H2-K^b^ or H2-D^b^ in MC-38 and B16-F10 cells to produce cells expressing a single classical MHC-I allele. Flow cytometric analysis and western blotting revealed that deletion of one classical H2 allele did not affect the expression of the other H2 molecule (**Supplementary Figures 2B–H**). Cells deleted of β2m were also produced as a negative control, which completely impaired expression of both H2-K^b^ and H2-D^b^ (**Supplementary Figures 2B–H**), as expected.

When mouse TAPBPR was immunoprecipitated from the cells expressing the single classical MHC-I alleles, strong bands for both the MHC-I heavy chain and β2m were observed in cells expressing H2-D^b^ (**Figure 2F**). In contrast, the interaction between TAPBPR and H2-K^b^ was more difficult to detect, with only a weak band detected for β2m in the H2-K^b^ expressing cells (**Figure 2F**). Immunoprecipitation using H2-D^b^ and -K^b^ specific antibodies confirmed that the anti-human MHC-I used in immunoblotting detects both H2-D^b^ and -K^b^ to a similar level, and both MHC-I molecules were able to pull down β2m under the experimental conditions used (**Supplementary Figure 2J**). Together, these results suggest that mouse TAPBPR exhibits increased binding to H2-D^b^ compared to H2-K^b^ in both MC-38 and B16 cells.

### 3.5 Analysis of the MHC-I immunopeptidome in B16 cells suggests TAPBPR restricts the peptide repertoire on H2-D^b^

**Figure 3:**
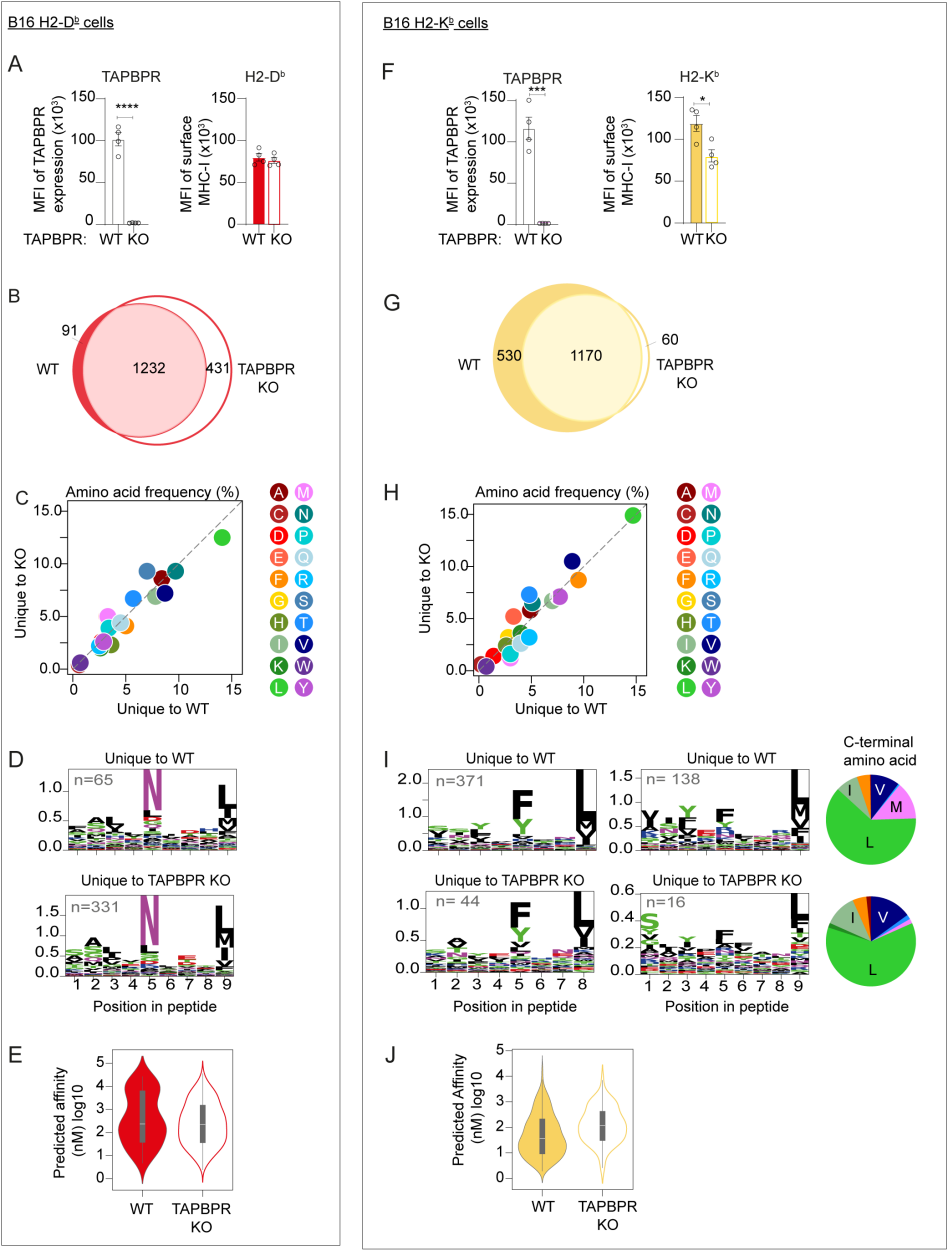
Effect of loss of TAPBPR on MHC-I surface expression and immunopeptidome on B16 cells expressing either H2-D^b^ or H2-K^b^. **(A, F)** Flow cytometric analysis showing efficiency of TAPBPR knockout on IFNγ-treated B16 cells expressing the single classical H2 alleles and the surface expression of either **(A)** H2-D^b^ or **(F)** H2-K^b^ on these B16 cells in the presence (WT) or absence (KO) of TAPBPR. Error bars show MFI -/+ SEM. Four independent experiments are shown. The asterisks (*) denote statistical difference between cell variants (*p ≤ 0.05, ***p ≤ 0.001, ****p ≤ 0.0001). **(B–E)** H2-D^b^ and **(G–J)** H2-K^b^ ligandomes from WT and TAPBPR KO IFNγ-treated B16 cells expressing single classical H2 alleles. **(B, G)** Venn diagrams show the size and overlap of the peptides eluted on **(B)** H2-D^b^ and **(G)** H2-K^b^. **(C, H)** Frequency (%) of each amino acid presented on peptides unique to WT or TAPBPR KO cells from **(C)** H2-D^b^ and **(H)** H2-K^b^. **(D, I)** SeqLogos showing the consensus sequences of 8mer (H2-K^b^ only) and 9mer (both H2-D^b^ and -K^b^) peptides unique to WT and TAPBPR KO cells, with pie charts showing the frequency of amino acids at the C-terminus for all unique peptides for H2-K^b^. **(E, J)** Violin plots show the NetMHC-predicted affinities of the peptides unique to TAPBPR WT and TAPBPR KO cells for **(E)** H2-D^b^ and **(J)** H2-K^b^. Statistics obtained using a Mann-Whitney U test.

Next, we examined the role mouse TAPBPR played in shaping the peptide repertoire on mouse MHC-I by comparing the immunopeptidome of cells with and without TAPBPR (**Supplementary Table 1**). Cells expressing the single classical H2 alleles were chosen for this work to avoid biasing peptide allocation to a specific H2 molecule based on affinity. On B16 cells expressing H2-D^b^ only, when TAPBPR was knocked out, the surface expression of H2-D^b^ remained unchanged (**Figure 3A**). This suggests that in B16 cells, H2-D^b^ is not dependent on TAPBPR for surface expression. Analysis of the peptidome demonstrated that while the majority of presented peptides on B16 cells were unaffected by TAPBPR expression, populations of peptides that were uniquely presented in the presence of TAPBPR (i.e. those potentially loaded by TAPBPR) and the absence of TAPBPR (i.e. those seemingly removed or prevented from loading by TAPBPR) were identified. For the H2-D^b^ expressing B16 cells, 91 peptides were uniquely identified in the presence of TAPBPR, while 431 were uniquely identified in the TAPBPR knockout cells (**Figure 3B**). As a broader immunopeptidome was presented in cells lacking TAPBPR, the presence of TAPBPR appears to restrict the overall peptide repertoire presented on H2-D^b^ on B16 cells, as has been observed previously for human TAPBPR on human MHC-I molecules (9, 21). No striking effects were observed on the amino acid frequency (**Figure 3C**), nor in the SeqLogo analysis (**Figure 3D**) or in the predicted affinity (**Figure 3E**) of peptides uniquely presented in the presence and absence of TAPBPR.

### 3.6 Analysis of the H2-K^b^ immunopeptidome on B16 cells hints at potential changes in the C-terminal anchor

On B16 cells expressing H2-K^b^ only, the deletion of TAPBPR resulted in the reduction of H2-K^b^ surface expression by approximately 32% (**Figure 3F**). This suggests that in B16 cells, H2-K^b^ is partially dependent on TAPBPR for its surface expression. Immunopeptidomic analysis revealed that 530 peptides were uniquely found in the presence of TAPBPR compared to only 60 peptides uniquely found in the TAPBPR knockout cells on H2-K^b^ (**Figure 3G**). This likely reflects the reduced H2-K^b^ expression on B16 cells in the absence of TAPBPR. While analysis of the amino acid frequency did not reveal any striking differences (**Figure 3H**), Seqlogo analysis of the peptides revealed the presence of a C-terminal methionine in some of the peptides unique to cells expressing TAPBPR (13.6%), which was lower in the peptides unique to cells lacking TAPBPR expression (1.7%) (**Figure 3I**; **Supplementary Table 2**). However, as the number of unique peptides was very low in the TAPBPR knockout cells, particularly when separated into 8-mers and 9-mers, it is difficult to draw firm conclusions from this data, as well as in relation to the predicted affinity of the unique peptides (**Figure 3J**).

### 3.7 Analysis of the MC-38 MHC-I immunopeptidome suggests TAPBPR restricts the peptide repertoire on H2-D^b^

**Figure 4:**
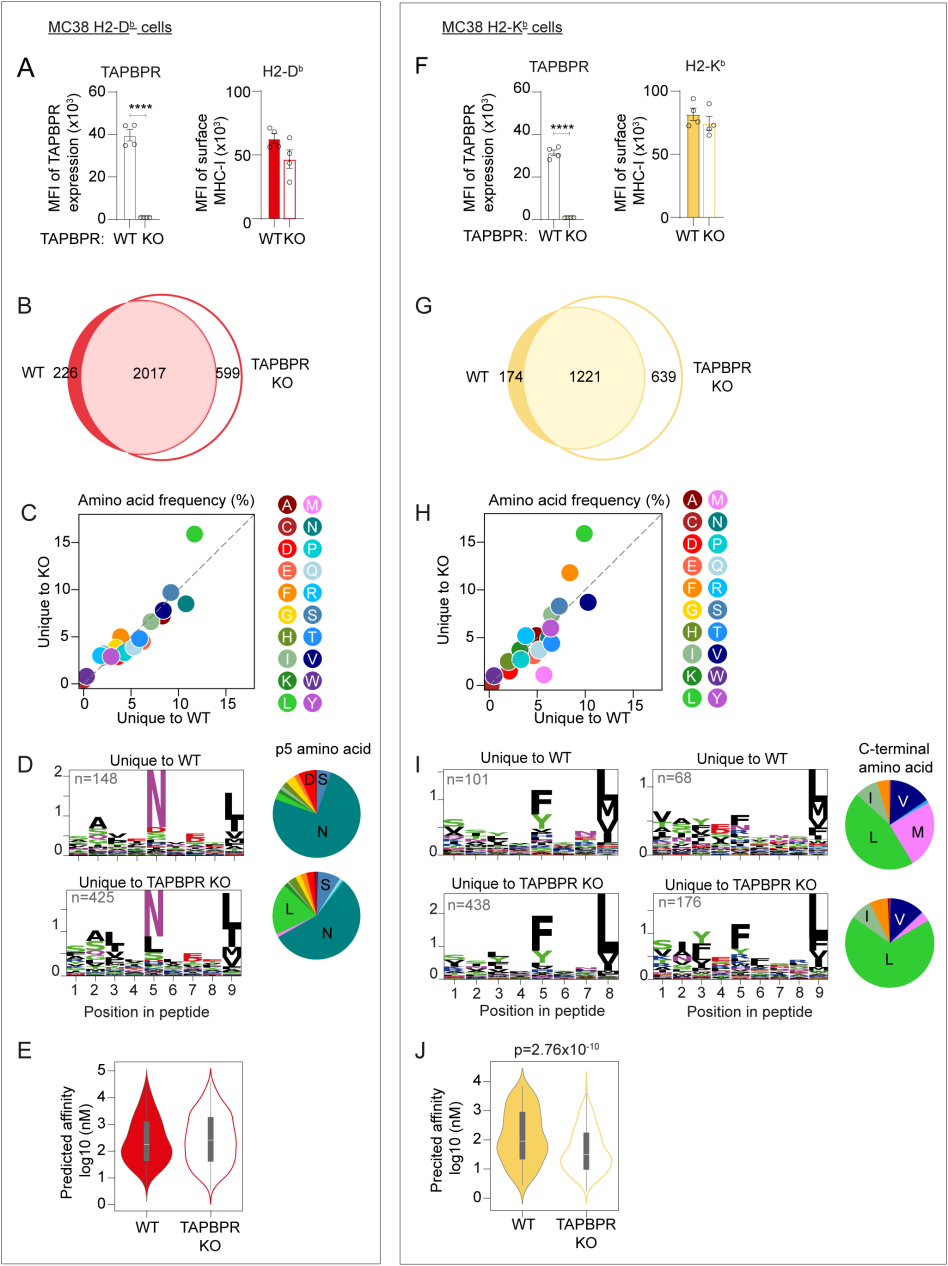
Effect of loss of TAPBPR on MHC-I surface expression and immunopeptidome on MC-38 cells expressing either H2-D^b^ or H2-K^b^. **(A, F)** Flow cytometric analysis showing efficiency of TAPBPR knockout on IFNγ-treated MC-38 cells expressing the single classical H2 alleles and the surface expression of either **(A)** H2-D^b^ or **(F)** H2-K^b^ on these B16 cells in the presence (WT) or absence (KO) of TAPBPR. Error bars show MFI -/+ SEM. Four independent experiments are shown. The asterisks (*) denote a statistical difference between cell variants (****p ≤ 0.0001). **(B–E)** H2-D^b^ and **(G–J)** H2-K^b^ ligandomes from WT and TAPBPR KO IFNγ-treated MC-38 cells expressing single classical H2 alleles. **(B, G)** Venn diagrams show the size and overlap of the peptides eluted on **(B)** H2-D^b^ and **(G)** H2-K^b^. **(C, H)** Frequency (%) of each amino acid presented on peptides unique to WT or TAPBPR KO cells from **(C)** H2-D^b^ and **(H)** H2-K^b^. **(D, I)** SeqLogos showing the consensus sequences of 8mer (H2-K^b^ only) and 9mer (both H2-D^b^ and -K^b^) peptides unique to WT and TAPBPR KO cells, with pie charts showing the frequency of amino acids at p5 for H2-D^b^ and at the C-terminus for H2-K^b^ for all unique peptides. **(E, J)** Violin plots show the NetMHC-predicted affinities of the peptides unique to TAPBPR WT and TAPBPR KO cells for **(E)** H2-D^b^ and **(J)** H2-K^b^. Statistics obtained using a Mann-Whitney U test.

Given the reduced expression of H2-K^b^ on the B16 cells in the absence of TAPBPR, we subsequently explored the effect of deleting TAPBPR on MHC-I in additional mouse cell lines. On MC-38 cells expressing the single classical H2 alleles, knockout of TAPBPR had no significant effect on total H2-D^b^ surface expression (**Figure 4A**). A similar finding was observed in MEF-BL/6–1 cells (**Supplementary Figure 3**). This suggests that H-2D^b^ is not dependent on TAPBPR for surface expression in IFNγ-treated MC-38 or MEF cells. Comparison of the H2-D^b^ peptide repertoire MHC-I on MC-38 cells with and without TAPBPR revealed 226 peptides were uniquely identified in the presence of TAPBPR, while 599 were uniquely identified in the TAPBPR knockout cells on H2-D^b^ molecules (**Figure 4B**; **Supplementary Table 1**). As a broader peptidome was presented in cells lacking TAPBPR, the presence of TAPBPR appears to restrict the overall peptide repertoire presented on H-2D^b^ on MC-38 cells, as was observed for H2-D^b^ on B16 cells (**Figure 3**). In MC-38 cells, TAPBPR absence had no obvious effect on the length of peptides presented on H2-D^b^ or -K^b^ (**Supplementary Figure 4**). Comparison of the peptides unique to TAPBPR-expressing and knockout MC-38 cells revealed an increase in the frequency of leucine (L) in peptides unique to TAPBPR knockout cells on H2-D^b^ molecules (**Figure 4C**). SeqLogo analysis of the unique peptides revealed an increase in leucine located at the anchor residue found at position 5 of 9-mer peptides unique to the TAPBPR knockout cells (2.7% in WT versus 19% in KO) (**Figure 4D**; **Supplementary Table 2**). No change in the overall predicted affinity was observed between the unique peptides presented in the presence and absence of TAPBPR (**Figure 4E**).

As TAPBPR not only affects the presence and absence of peptides, we also assessed TAPBPR-mediated changes in peptide abundance using label-free quantitation (**Supplementary Figure 5**). Analysis on these enriched peptides confirmed that the loss of TAPBPR enhanced the presentation of peptides containing leucine residues (**Supplementary Figure 5B**), with p3, p5 and p9 of the 9-mer peptides all showing enhanced presentation of leucine (**Supplementary Figure 5C**). No change in the overall predicted affinity was observed between the peptides enriched in the presence or the absence of TAPBPR (**Supplementary Figure 5D**).

### 3.8 In MC-38 cells, mouse TAPBPR promotes the loading of peptides containing a C-terminal methionine on H2-K^b^

On MC-38 cells expressing H2-K^b^ only, knockout of TAPBPR did not alter surface expression of H2-K^b^ (**Figure 4F**). A similar finding was observed for H2-K^b^ on MEF-BL/6–1 cells (**Supplementary Figure 3**). This suggests that H2-K^b^ is not dependent on TAPBPR for surface expression in MC-38 or MEFs, in contrast to the observation for H2-K^b^ on the B16 TAPBPR knockout cells. Thus, there seems to be some cell line variability in the dependency of mouse MHC-I on mouse TAPBPR.

Immunopeptidomic analysis of the H2-K^b^ expressing MC-38 cells identified 174 peptides uniquely found in the presence of TAPBPR compared to 639 uniquely found in the TAPBPR knockout cells (**Supplementary Table 1**; **Figure 4G**). This suggests that in MC-38 cells, TAPBPR plays a restrictive role on the peptidome on H2-K^b^. Comparison of the unique peptides suggested an increase in the frequency of methionine (M) in peptides unique to TAPBPR-expressing cells and an increase in the frequency of leucine (L) in the peptides unique to TAPBPR-knockout cells (**Figure 4H**). SeqLogo analysis of the unique peptides revealed that the altered expression of the amino acids was predominantly located at the C-terminus of peptides, with the peptides unique to TAPBPR-expressing cells containing more C-terminal methionines (24.7%) compared to peptides unique to the TAPBPR knockout cells (3.44%) (**Figure 4I**; **Supplementary Table 2**). This finding aligns with observations in the B16 dataset (**Figure 3I**). Analysis of the TAPBPR-mediated changes in peptide abundance using label-free quantitation confirmed that TAPBPR presence promoted the presentation of peptides containing a C-terminal methionine on H-2K^b^ (**Supplementary Figure 5**). Taken together, these findings suggest that TAPBPR promotes the loading of peptides containing a C-terminal methionine onto H2-K^b^. Surprisingly, the peptides unique to the TAPBPR-expressing cells appeared to exhibit a lower predicted affinity than those unique in TAPBPR-deficient cells (**Figure 4J**), suggesting that the loading of the peptides containing the C-terminal methionine may be potentially of weaker affinity for H2-K^b^.

### 3.9 Recombinant soluble mouse TAPBPR is an inefficient exchange catalyst on plasma membrane-expressed mouse MHC-I molecules

Having confirmed that mouse TAPBPR shapes the immunopeptidome on both H2-D^b^ and -K^b^, we attempted to explore its ability to catalyze peptide exchange on these two MHC-I molecules. Previously, we have shown that recombinant soluble human TAPBPR can perform peptide exchange on plasma membrane-expressed MHC-I (21, 33, 35). Therefore, we initially attempted to use soluble recombinant mouse TAPBPR in our peptide exchange assays. While differential scanning fluorimetry confirmed protein stability (**Supplementary Figure 6A**), we found recombinant soluble mouse TAPBPR was unable to efficiently bind to surface MHC-I molecules on MC-38 cells (**Supplementary Figure 6B**), in contrast to human TAPBPR, which could (**Supplementary Figure 6C**). Correspondingly, recombinant soluble mouse TAPBPR was unable to perform peptide exchange on mouse MHC-I expressed on MC-38 cells (**Supplementary Figure 6D**) while recombinant soluble human TAPBPR promoted peptide exchange on both H2-D^b^ and H2-K^b^ (**Supplementary Figure 6E**). These results suggest recombinant soluble mouse TAPBPR likely has a lower affinity for MHC-I than recombinant human TAPBPR, in line with findings from Sun et al. (32*)*,.

### 3.10 Endogenously expressed TAPBPR is barely detectable on the plasma membrane of MC-38 cells

**Figure 5:**
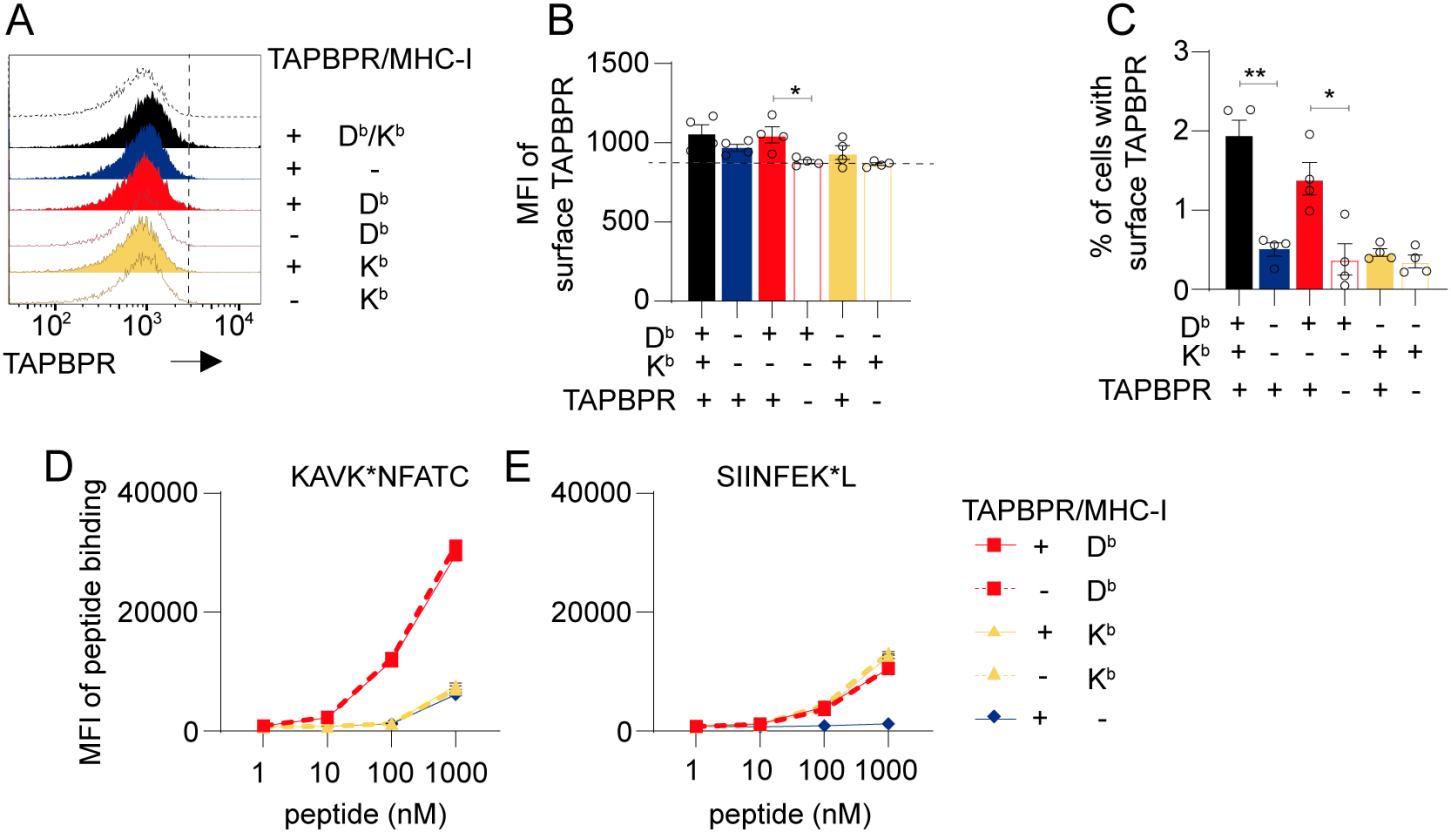
Endogenously expressed TAPBPR is barely detectable on the plasma membrane of MC-38 cells. **(A–C)** Mouse TAPBPR is barely detectable on the plasma membrane of MC-38 cells treated with IFNγ. **(A)** Histograms and **(B)** bar charts showing MFI of surface TAPBPR and **(C)** % of cells potentially positive for TAPBPR on WT MC-38 (black filled) and variants lacking β2m (blue filled), expressing H2-D^b^ only (red filled) or H2-K^b^ only (yellow filled). TAPBPR-deficient variants of cells expressing H2-D^b^ only (open red) or H2-K^b^ only (open yellow) are included as controls. Events to the right-hand side of the dashed line in A indicate cells considered surface TAPBPR positive in **(C)**. The dashed line in B represents the background staining observed on these cells, which is not due to TAPBPR. **(D, E)** Deletion of mouse TAPBPR does not affect the loading of peptide onto plasma membrane-expressed H2-D^b^ or H2-K^b^. IFNγ-treated MC-38 cells expressing either H2-D^b^ (red) or H2-K^b^ (yellow) with endogenous TAPBPR (solid line) or with TAPBPR knocked out (dashed line) were pulsed with 1–1000 nM **(D)** KAVK*NFATC peptide or **(E)** SIINFEK*L peptide for 30 min, with TARMA labelled peptide binding measured using flow cytometry. Cells lacking surface expression of H2-D^b^/K^b^ (β2 m-deficient variant)(blue line) are included as a negative control. Statistics obtained using ordinary one-way ANOVA with Tukey’s multiple comparison.

Previously, we reported that when human TAPBPR is overexpressed in HeLaM cells, it traffics to the plasma membrane with MHC-I and can be used for peptide loading on MHC-I (21, 35). As endogenous mouse TAPBPR has been reported to be present at the plasma membrane of some tumor cell lines (34), we sought to explore whether MC-38 cells expressed TAPBPR on their cell surface. TAPBPR was barely detectable on MC-38 cells (**Figure 5A**). Only a very slight decrease in MFI was observed on TAPBPR knockout cells compared to their WT counterparts for MC-38 cells expressing the single classical H2 alleles (**Figure 5B**). Furthermore, only a few percent of the MC-38 population expressed surface TAPBPR (**Figure 5C**). The small amount of plasma membrane-expressed TAPBPR appeared to be in complex with MHC-I, as indicated by its reduction upon β2m deletion (**Figure 5C**, blue line). However, peptide pulsing experiments also revealed no change in peptide loading onto H2-D^b^ or K^b^ in the presence or absence of TAPBPR on MC-38 cells (**Figure 5D**). This supports the notion that TAPBPR is not highly expressed on these cells, even after IFNγ induction. While the immunopeptidomics data suggested that peptides uniquely presented on MHC-I in the presence of TAPBPR had reduced affinity (**Figure 4**), the peptide pulse data suggest there is no obvious change in the overall peptide receptivity of MHC-I on MC-38 cells in the presence or absence of TAPBPR (**Figure 5D**).

### 3.11 Overexpressed mouse TAPBPR traffics to the plasma membrane with MHC-I in MC-38 cells

**Figure 6:**
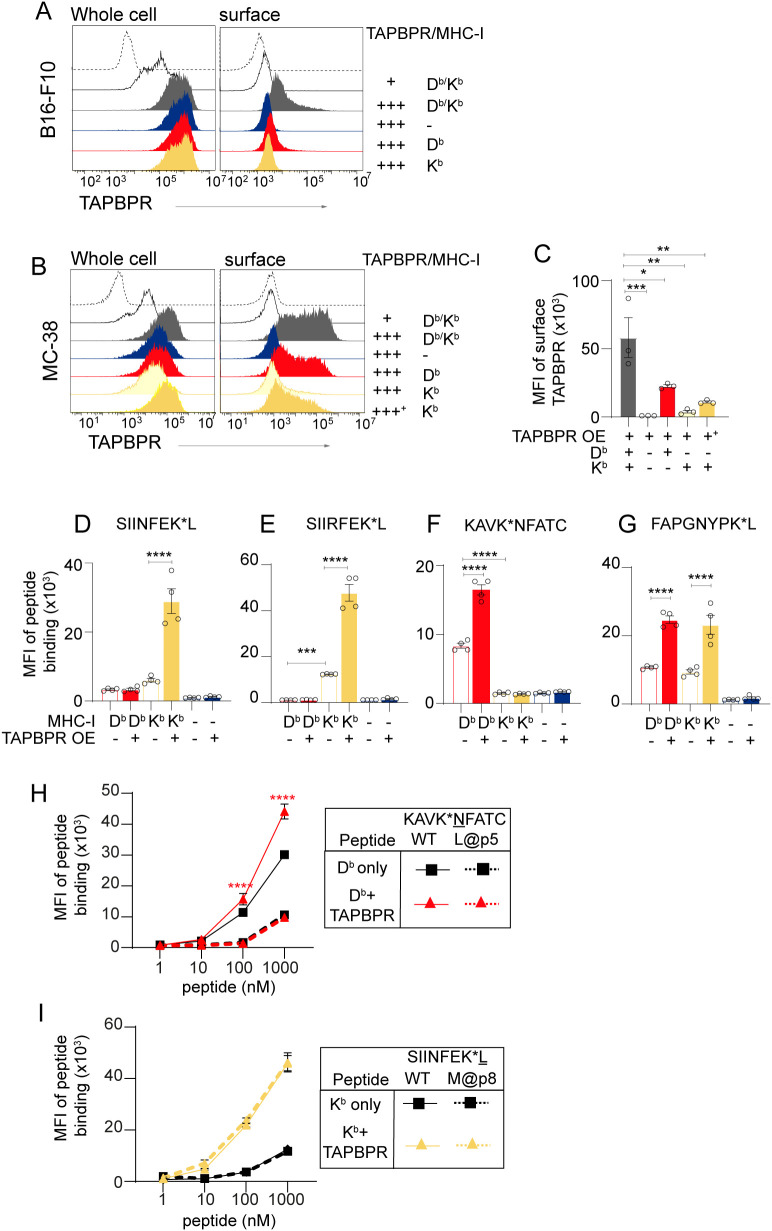
Mouse TAPBPR can promote peptide loading on both H-2D^b^ and H2-K^b^. **(A–C)** Overexpression of mouse TAPBPR results in its plasma membrane localization. Representative histograms showing whole cell and surface expression of TAPBPR on **(A)** B16 and **(B)** MC-38 cells overexpressing mouse TAPBPR. TAPBPR was overexpressed in WT cells (grey filled), β2m knockout cells (blue filled), cells expressing H2-D^b^ only (red filled) and cells expressing H2-K^b^ only (yellow filled). In B, MC-38 cells expressing H2-K^b^ only were sorted for high surface TAPBPR expression (+++^+^). Unstained cells (dashed line) and WT cells expressing endogenous TAPBPR (black line) are included as controls. **(C)** Bar graph showing mean fluorescence intensity (MFI) of surface TAPBPR on the MC-38 cell line panel overexpressing mouse TAPBPR from 3 independent experiments. Error bars represent -/+ SEM. **(D–G)** TAPBPR-mediated peptide loading on MHC-I. Bar graphs show MFI of **(D)** SIINFEK*L, **(E)** SIIRFEK*L, **(F)** KAVK*NFATC, or **(G)** FAPGNYPK*L onto MC-38 cells expressing H2-D^b^ only (H2-K^b^ knockout), H2-K^b^ only (H2-D^b^ knockout) or lacking both H2-D^b^ and -K^b^ (β2m knockdown cells) in the absence (-) and presence (+) of surface mouse TAPBPR when treated with 100 nM of TAMRA labelled peptide binding peptide for 30 minutes. **(H, I)** Line graphs showing the ability of plasma-membrane-expressed mouse TAPBPR to load anchor-modified peptides onto MHC-I. **(H)** Binding of KAVK*NFATC (solid line) or its derivative KAVK*LFATC (dashed line) onto MC-38 cells expressing H2-D^b^ only -/+ surface TAPBPR. **(I)** Binding of SIINFEK*L (solid line) or its derivative SIINFEK*M (dashed line) onto MC-38 expressing H2-K^b^ only -/+ surface TAPBPR. Cells were treated with 1–1000 nM TARMA labelled peptide for 30 min. Error Bars indicate -/+ SEM. Data from two independent experiments, each in duplicate, are shown. Statistics obtained using ordinary one-way ANOVA with Tukey’s multiple comparison.

In a final attempt to develop cell-based assays to test the peptide-loading potential of mouse TAPBPR, we overexpressed the full-length protein in our various mouse cell lines and assessed its plasma membrane expression. This revealed some significant differences between the cell lines. In WT B16 cells, overexpressed mouse TAPBPR was detectable at low levels on the cell surface, despite high levels of TAPBPR overexpression being confirmed using permeabilized cells (**Figure 6A**, grey line). In contrast, in WT MC-38 cells, which express both H-2D^b^ and K^b^ at physiological levels, overexpressed mouse TAPBPR was present at very high levels on the cell surface (**Figures 6B, C**, grey line/bar). High levels of surface TAPBPR upon overexpression were also observed on MEF cells (**Supplementary Figure 7**). This suggests potential differences in the intracellular retention and/or export pathways for TAPBPR-MHC-I complexes in MC-38 and B16 cells.

When mouse TAPBPR was overexpressed in cell lines lacking β2m expression, TAPBPR was not detectable on the plasma membrane on B16, MC-38 or MEF, despite being expressed at high levels intracellularly (**Figures 6A–C**; **Supplementary Figure 7**, blue line/bar). This suggests that surface expression of overexpressed TAPBPR is dependent on its ability to interact with MHC-I. To explore this further, TAPBPR was overexpressed in our cell lines expressing the single classical MHC-I alleles. In MC-38 expressing H2-D^b^ only, overexpressed TAPBPR was efficiently trafficked to the plasma membrane (**Figures 6B, C**, red line/bar). In contrast, in MC-38 with H2-K^b^ only, overexpressed TAPBPR was detectable only at low levels (**Figures 6B, C**, pale yellow line). A similar pattern of TAPBPR dependence on MHC-I for surface expression was observed in MEF cells (**Supplementary Figure 7**). These results suggest that TAPBPR: H2-D^b^ complexes may form a stable structure that is capable of passing ER quality control checkpoints and/or that associates together long enough to be exported through the secretory pathway to the plasma membrane. In contrast, the majority of TAPBPR: H2-K^b^ complexes are likely more transient in nature and/or dissociate before entering the secretory pathway. That said, when we subsequently sorted MC-38 H2-K^b^+/TAPBPR overexpressed cells for high surface TAPBPR, we were able to enrich for cells expressing surface TAPBPR: H-2K^b^ complexes (**Figures 6B, C**, dark yellow line/bar). Thus, the overexpression of mouse TAPBPR in MC-38 cells provided a model system to subsequently explore TAPBPR’s ability to mediate peptide loading onto both H2-D^b^ and -K^b^ in our cell-based assays.

### 3.12 Mouse TAPBPR can promote peptide loading on both H2-D^b^ and H2-K^b^

Finally, we tested the ability of plasma membrane-expressed TAPBPR to perform peptide loading on both H2-D^b^ and -K^b^ on MC-38 overexpressing TAPBPR. TAMRA-labelled peptides were tested for their ability to be loaded onto both H2-D^b^ and -K^b^ using mouse TAPBPR. Our results demonstrated that mouse TAPBPR was very efficient at promoting the loading of SIINFEK*L and SIIRFEK*L (a mutant of SIINFEKL with increased specificity for H2-K^b^) onto H2-K^b^ molecules but did not promote the loading of these peptides onto H2-D^b^ (**Figures 6D, E**). Mouse TAPBPR was also capable of increasing the loading of KAVK*NFATC onto H2-D^b^ but not H2-K^b^ (**Figure 6F**). The loading of FAPGNYPK*L was also enhanced in the presence of mouse TAPBPR, on both the MHC-I molecules (**Figure 6G**). These data demonstrate that mouse TAPBPR can perform peptide loading on both H2-D^b^ and H2-K^b^.

### 3.13 Mouse TAPBPR efficiently loads a peptide containing a C-terminal methionine onto H2-K^b^

As the immunopeptidomic analysis suggested that mouse TAPBPR expression decreased the presentation of peptides containing a leucine at position 5 from H2-D^b^ and increased the presentation of peptides containing a C-terminal methionine onto H2-K^b^ (**Figure 4**; **Supplementary Figure 5**), we sought to directly test the ability of TAPBPR to achieve this in our peptide loading assays. To this end, we made a derivative of TARMA labelled KAVK*NFATC peptide replacing the asparagine at position 5 with a leucine residue (KAVK*LFATC) and a variant of the SIINFEK*L peptide in which the C-terminal leucine residue was replaced with a methionine (SIINFEK*M). We then tested the ability of mouse TAPBPR to load these peptides onto H2-D^b^ and -K^b^. While TAPBPR was efficient at loading the canonical peptide KAVK*NFATC onto H2-D^b^, it was not able to load the derivative peptide KAVK*LFATC onto this MHC-I molecule (**Figure 6H**, red lines). Although this finding aligns with the results of our immunopeptidomic studies, further analysis indicated that KAVK*LFATC has a significantly reduced affinity for H2-D^b^ (**Figure 6H**), making it difficult to draw clear conclusions using this peptide in this loading assay. For H2-K^b^, both SIINFEK*L and SIINFEK*M exhibited a similar propensity to bind to this MHC-I in the absence of TAPBPR (**Figure 6I**, black lines). We found that surface mouse TAPBPR promoted the loading of SIINFEK*M as efficiently as SIINFEK*L onto H2-K^b^ (**Figure 6I**, yellow lines). Thus, demonstrating directly that mouse TAPBPR can load peptides containing a C-terminal methionine onto H2-K^b^, supporting our immunopeptidomic findings.

## 4 Discussion

Here, we characterized the functional properties of mouse TAPBPR by exploring its biology in cell lines derived from C57BL/6 mice, which express only two classical MHC-I molecules. Our data demonstrate that mouse TAPBPR binds both H2-D^b^ and H2-K^b^. Akin to the allelic binding preference of human TAPBPR for HLA-A, over HLA-B and -C molecules (33), we found it easier to detect the association of mouse TAPBPR with H2-D^b^ than with H2-K^b^ (**Figure 2**). While we did not determine the affinity of mouse TAPBPR with either MHC-I here using surface plasmon resonance, our findings suggest that within a complex cellular environment, the H2-D^b^: mouse TAPBPR association is potentially more prolonged than the H2-K^b^: mouse TAPBPR interaction. In relation to this, human TAPBPR has been crystallized with H2-D^b^, as well as H2-D^d^ (23, 26). However, no structural information is available yet for TAPBPR in complex with H2-K^b^. Our inability to get recombinant soluble mouse TAPBPR to bind to H2-D^b^ or -K^b^ on the plasma membrane fits with the previous suggestion that mouse TAPBPR has a weaker affinity for MHC-I compared to human TAPBPR (32).

Because TAPBPR expression, along with MHC-I and other dedicated components of the antigen presentation pathway, is upregulated by IFNγ, we conducted all experiments on IFNγ-treated cells. We acknowledge that IFNγ treatment can alter cells in many crucial ways. For example, IFNγ treatment can lead to a tryptophan shortage by increasing indoleamine 2,3-dioxygenase 1 expression, resulting in tryptophan-to-phenylalanine codon reassignment (W>F) during protein synthesis, thereby altering the landscape of antigens presented on MHC-I molecules (48). However, as B16-F10 cells have barely any MHC-I expressed (49, 50) and lack functional peptide loading complexes, due to the absence of TAP and tapasin expression at a basal level (51), we deemed it necessary to use IFNγ-treated cells for this work.

Immunopeptidomic analysis confirmed that mouse TAPBPR shapes the peptide repertoire presented on mouse MHC-I molecules (**Figures 3**, **4**), as is already known for the human ortholog (9, 19, 21, 22). In our previous work using cell lines expressing multiple MHC-I alleles, we did not observe a clear effect of TAPBPR on MHC-I peptide-binding motifs. However, our recent switch to cells expressing a single classical MHC-I for immunopeptidomic analysis is allowing us to gain more insight into precisely how TAPBPR affects the immunopeptidome. Recently, our characterization of cells expressing only HLA-B44 molecules has revealed a role for TAPBPR in promoting the loading of specific amino acids at the C-terminus of peptides (52). In the case of HLA-B44, human TAPBPR promoted the loading of peptides containing a C-terminal tryptophan residue (52). Here, we similarly find that in TAPBPR-expressing cells there is an increase in the presentation of peptides containing a specific C-terminal residue: a methionine for H2-K^b^ molecules (**Figures 3**, **4**). Furthermore, we demonstrate that mouse TAPBPR can load peptides containing a C-terminal methionine on H2-K^b^ molecules (**Figure 6**). Thus, we are now beginning to gain a deeper understanding of the selection criteria TAPBPR imposes on the peptide repertoire. Intriguingly, the MHC-I allotypes we have observed TAPBPR promoting the loading of a specific residue at the C-terminus are ones that are more difficult to detect in association with TAPBPR. One can speculate that this may be due to faster off-rates of these allotypes from TAPBPR due to the incoming peptide efficiently outcompeting TAPBPR for MHC-I binding. While loading of peptides with specific C-termini will be dependent on properties of the MHC-I molecule itself, differences in the editing loops between human and mouse TAPBPR, such as the leucine versus a phenylalanine in the loop at position 30 (**Figure 1**), may also contribute to the selection of specific residues at the C-terminus.

TAPBPR presence promoted the presentation of a C-terminal methionine on H2-K^b^ in both B16-F10 and MC-38 cell lines. Furthermore, the overall restrictive effect of TAPBPR on the H2-D^b^ peptide repertoire was consistent between the two cell lines. However, we observed notable differences in how TAPBPR shaped the size of the H2-K^b^ immunopeptidome between the two cell lines: TAPBPR presence restricted the H2-K^b^ peptide repertoire in IFNγ-treated MC-38 cells, but surprisingly expanded it in IFNγ-treated B16-F10 cells. The reason for this difference between the two cell lines is unclear. However, our flow cytometry (**Figures 2F**, **3F**) and western blot data (**Supplementary Figure 2H**) suggest that TAPBPR expression is higher in IFNγ-treated B16-F10 cells compared to IFN-γ-treated MC38 cells (2.5-fold higher based on MFI in **Figures 2F**, **3F**). This higher expression may contribute to the more severe effect of TAPBPR deletion on B16-F10 H2-K^b^ surface expression and, consequently, the immunopeptidome. The stability of ER-resident H2-K^b^ molecules may also differ between the two cell lines. MC-38 cells have high basal levels of MHC-I, tapasin and TAPBPR compared to B16-F10 cells (**Supplementary Figure 2H****).** The addition of IFNγ to MC-38 cells results in a comparatively modest increase in the antigen presentation machinery and the pathway appears maximized, enabling efficient H2-K^b^ peptide loading. In this context, TAPBPR is not essential for H2-K^b^ stabilization and performs a role in monitoring the peptide-loading status of H2-K^b^ molecules before ER exit. In contrast, in B16-F10 cells, IFNγ treatment induces a massive increase in both MHC-I transcription and translation from an extremely low basal level (49) (**Supplementary Figure 2H**), which may conceivably transiently overwhelm the peptide-loading and quality-control machinery. Furthermore, while individual components of the MHC-I pathway are typically maximally expressed following 48 hours of IFNγ treatment, the coordinated assembly of fully functional peptide-loading complexes may occur more slowly in B16-F10 cells than in MC-38 cells. Thus, we speculate that in B16-F10 cells, there is a significant population of kinetically unstable, peptide-receptive H2-K^b^ molecules that require stabilization via transient engagement with TAPBPR. TAPBPR may then assist these peptide-receptive MHC-I molecules to incorporate into the PLC for peptide acquisition. Alternatively, the high levels of TAPBPR in B16-F10 cells may enable it to access peptide-sufficient microenvironments to load H2-K^b^ molecules. Thus, TAPBPR presence promotes peptide acquisition in B16-F10 cells and knocking it out reduces H2-K^b^ surface expression. The reason for H2-K^b^ being more sensitive to TAPBPR loss than H2-D^b^ in B16-F10 cells may be due to differences in peptide repertoire breadth, given that H2-D^b^ appears to present a broader peptide repertoire than H2-K^b^ (53). Finally, differences in peptide repertoires available within the ER of the cell lines used may also contribution to the variation observed.

While UGGT1 has been shown to associate with human TAPBPR: MHC-I complexes (19, 20), it was not observed in association with mouse TAPBPR: MHC-I complexes in the three cell lines investigated here (**Figure 2**). The free cysteine residue in TAPBPR, which we had identified as being involved in associating with UGGT1, is conserved in mouse TAPBPR. Thus, we expected UGGT1 to be part of the mouse TAPBPR: MHC-I complex. One possible explanation for the lack of detection of UGGT1 association is the antibody used to isolate mouse TAPBPR in the pulldowns. While we screened the mouse TAPBPR antibodies generated for use in specific techniques, we did not perform epitope screening in this instance. Although the TAPBPR: MHC-I complex was detected with the antibody clone used here, a different clone might have enabled detection of UGGT1. Interestingly, while UGGT1-deficient MEF cells clearly demonstrated a role for UGGT1 in mouse MHC-I maturation and surface expression, a direct association with H2-D^b^ or H2-K^b^ was not demonstrated (54). Therefore, the interaction of UGGT1 with mouse MHC-I or mouse TAPBPR: MHC-I complexes may be more transient in nature and/or difficult to detect.

Instead of the UGGT1 association, an interaction between mouse TAPBPR and calnexin was intriguingly observed. Calnexin is a lectin-like ER chaperone that binds monoglucosylated N-linked glycans on nascent glycoproteins (55). Our results suggest that a direct interaction between calnexin and mouse TAPBPR can occur, as the association between the two proteins remained detectable, albeit at reduced levels, in β2m knockdown cells, which disrupts the TAPBPR: MHC-I interaction (**Figure 2**). As mouse TAPBPR contains N-linked glycans (**Figure 1B**; **Supplementary Figure 1B**), calnexin may well be involved in facilitating its initial folding. Previous work by Cresswell and colleagues also suggested a role for calnexin, in combination with ERp57, in generating an intermediate TAP-tapasin complex (56).

Our results here also support a complex between mouse TAPBPR and MHC-I associated with calnexin, which we have not observed for human TAPBPR. This is likely due to intrinsic properties of mouse MHC-I molecules, specifically their N-linked glycans. While all MHC-I molecules have an N-linked glycan at position 86, mouse MHC-I molecules have a second conserved N-linked glycan in the α2 domain, with some mouse MHC-I molecules also having a third glycan in the α3 domain (57, 58)(**Figure 1B**). Calnexin is known to chaperone free heavy chain forms of MHC-I via an association involving the N86-linked glycan (59). While for human MHC-I molecules calnexin is exchanged for calreticulin upon β2m (5), mouse MHC-I molecules remain associated with calnexin following β2m binding and only dissociate from calnexin when peptide is loaded (60). These differences are thought to be due to species-specific characteristics of the MHC-I heavy chain itself, as H2-K^b^ expressed in human cells remained associated with calnexin following β2m binding (5) and the introduction of a second glycan onto HLA-A*02:01 at position 176 not only increased its association with calnexin but also resulted in the detection of HLA-A*02:01/β2m/calnexin complexes (61). It has been proposed that while β2m association with MHC-I appears to displace calnexin bound to N86, it does not displace calnexin associated with other glycans on mouse MHC-I (N176 or N256). Thus, the observed differences between human and mouse TAPBPR complexes, specifically regarding UGGT1 versus calnexin association, are potentially not due to inherent differences in TAPBPR itself, but rather reflect intrinsic differences in the MHC-I molecules, particularly their glycosylation patterns, with the additional N-linked glycan in mouse MHC-I favoring calnexin engagement.

Although components of the peptide loading complex were found to associate with TAPBPR upon its overexpression, we were unable to detect these interactions at physiological TAPBPR expression levels (**Figure 2**). This raises questions about whether the association of mouse TAPBPR with the peptide loading complex is genuine or an experimental artefact observed with TAPBPR overexpression. In our initial characterization of human TAPBPR, we also observed its incorporation into the peptide loading complex upon its overexpression, but similarly, we were unable to detect the interaction under more physiological conditions (18).

It has previously been suggested that mouse TAPBPR is expressed on the cell surface, where it acts as a negative regulator of T cell function (34). Here, we found it difficult to detect significant amounts of mouse TAPBPR on the plasma membrane when expressed naturally under IFNγ induction, with no obvious change in mouse TAPBPR antibody binding to WT and TAPBPR knockout cells (**Figure 5**). Thus, there appear to be cellular control mechanisms inhibiting TAPBPR expression on the plasma membrane, which would be unusual for a protein with a bona fide role at the plasma membrane. Our work here demonstrates a role for endogenously expressed mouse TAPBPR in MHC-I peptide selection within an intracellular environment, as has been found for human TAPBPR. We did observe that overexpression of mouse TAPBPR in the C57BL/6 cell lines resulted in significant expression on the plasma membrane (**Figure 6**), as has been observed previously with human TAPBPR overexpressed in HeLa cells (35). This is possibly due to saturation of cellular retention machinery. However, given its dependence on MHC-I, the plasma membrane-localized mouse TAPBPR appears to be associated with MHC-I (**Figure 6**), rather than existing in a free, unbound state as previously suggested (34). Thus, the negative regulatory effects observed by Lin et al. could be due to TAPBPR blocking MHC-I recognition by T cells, rather than its association with an as-yet-unidentified T cell receptor (34).

Using C57BL/6-derived cell lines limited murine TAPBPR studies to two classical MHC-I molecules. Examination of TAPBPR’s effect on other murine MHC-I molecules may provide additional insight into its role in the immunopeptidome. Furthermore, research into TAPBPR’s roles across various cell types and its orthologues will clarify its potential in antigen presentation and immune regulation.

## Data Availability

The datasets presented in this study can be found in online repositories. The mass spectrometry proteomics data have been deposited to the ProteomeXchange Consortium via the PRIDE (62) partner repository with the dataset identifiers PXD068540 (mouse TAPBPR association partners) and PXD069965 (immunopeptidomes). The datasets for this study can be found in the PRIDE database [https://www.ebi.ac.uk/pride].
