# Supplementary material for: Mouse TAPBPR shows functional similarity to human TAPBPR in shaping the MHC-I immunopeptidome
Source: Front Immunol. 2026 Apr 15;17:1756668. doi: 10.3389/fimmu.2026.1756668 (PMC13124576; doi:10.3389/fimmu.2026.1756668)
Supplement: Supplementary file 1 [file DataSheet1.pdf]

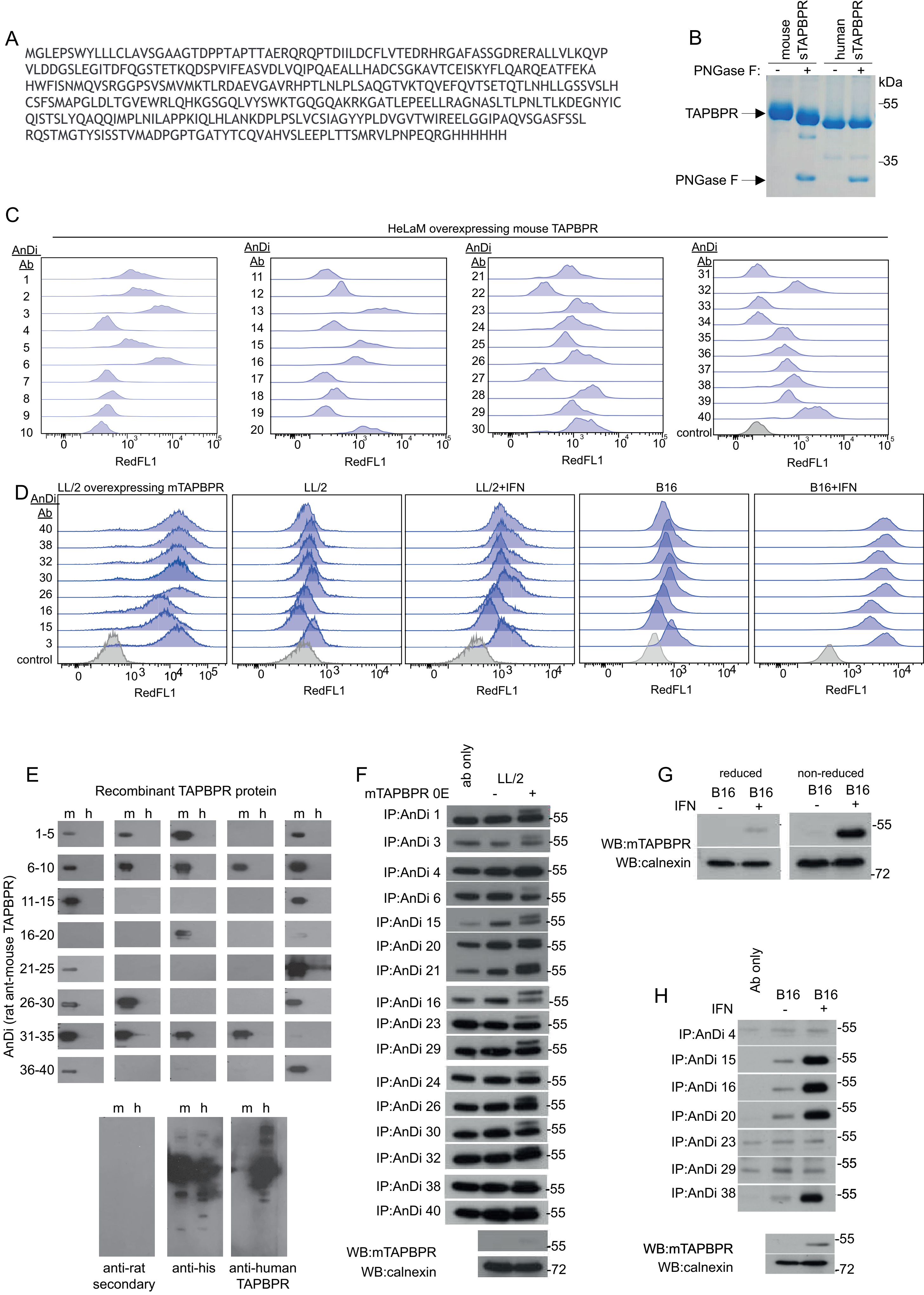

Supplementary Figure 1: Development of mouse TAPBPR specific monoclonal antibodies.

(**A**) Protein sequence of recombinant soluble mouse TAPBPR tagged at the C-terminus with 6xHis. (**B**) Production of recombinant soluble mouse TAPBPR. Coomassie gel on recombinant mouse TAPBPR and human TAPBPR following treatment -/+ PNGase F. Recombinant mouse TAPBPR was subsequently immunised into rats to produce anti-mouse-TAPBPR specific antibody series named AnDi. (**C**) Supernatant from the 40 AnDi rat hybridomas raised against mouse TAPBPR were tested for their ability to detect mouse TAPBPR in flow cytometry on permeabilized HeLa-M cells overexpressing mouse TAPBPR. (**D**) 8 Andi antibodies were further tested for their ability to work on permeabilized mouse cell lines. The following cell lines were used: LL/2 overexpressing mouse TAPBPR, LL/2, LL/2 treated with IFNγ to upregulate TAPBPR expression, B16 and B16 treated with IFNγ. (**E**) 40 AnDi antibodies were tested for their ability to detect recombinant soluble mouse TAPBPR (m) and recombinant soluble human TAPBPR (h) in western blotting with AnDi25 deemed as most efficient. In the bottom panel, blotting with the anti-rat secondary only is included as a negative control, while blotting with an anti-His antibody and for anti-human TAPBPR are included as positive controls. (**F**) Selected AnDi antibodies were tested for their ability to immunoprecipitate mouse TAPBPR from LL/2 cells and LL/2 cells overexpressing mouse TAPBPR. AnDi25 was used to detect mouse TAPBPR in western blot. (**G**) AnDi25 antibody is more efficient at detecting TAPBPR under non-reducing conditions compared to reducing conditions. Lysates from B16 cells -/+ IFNγ treatment were used. Blotting for calnexin is included as a loading control. (**H**) Selected AnDi antibodies were tested for their ability to immunoprecipitate mouse TAPBPR from B16 cells -/+IFNγ treatment. Samples were separated under non-reducing condition with TAPBPR detect via western blot using AnDi25. In E-G, blotting for calnexin is included as a loading control. Following this characterization of the rat-anti-mouse TAPBPR antibodies, AnDi3 was selected for flow cytometry, AnDi25 for immunoblotting, and AnDi38 for immunoprecipitation.

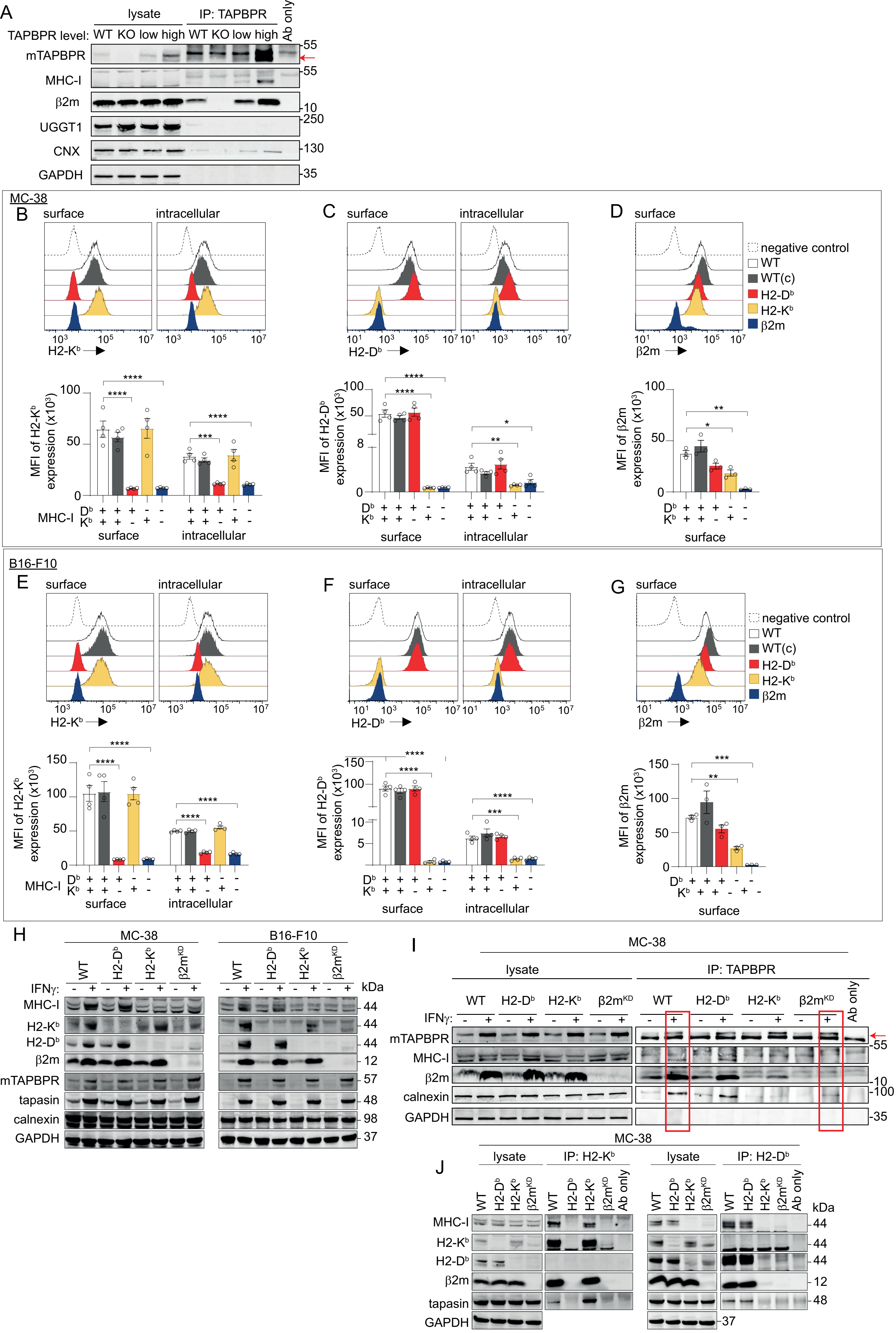

Supplementary Figure 2: Further confirmation of murine TAPBPR association partners and characterisation of MHC-I knockout cell lines.

(**A**) Murine TAPBPR immunoprecipitated from IFN-γ treated MC-38 cells pulls down calnexin but not UGGT1. In addition to WT and TAPBPR KO cells, MC-38 cells overexpressing low and high levels of transduced mouse TAPBPR are included as positive controls. Representative histograms and bar graphs showing mean fluorescence intensity (MFI) of surface and intracellular (**B,E**) H-2K^b and (**C,F**) H-2D^b expression and (**D,G**) surface β2m expression for (**B-D**) MC-38 and (**E-G**) B16-F10 on the following cell variants; WT, WT control (WT(c)) subjected to RNP method with a negative RNA guide, cells expressing H-2D^b only (H2-K^b knockout) or H-2K^b only (H2-D^b knockout) and cells with β2m expression knocked down. Isotype controls are included for H2 staining, while staining with second antibody only are included as negative controls for staining for TAPBPR and β2m. Error bars indicate -/+ SEM. Three or four independent experiments are shown. Statistics obtained using ordinary one-way ANOVA with Tukey's multiple comparison. **(H)** Immunoblots confirm the knockout of MHC-I following CRISR-Cas9. Immunoblots indicating abundance of total MHC-I, H-2K^b, H-2D^b, β2m, mouse TAPBPR (mTAPBPR), tapasin, calnexin, and GAPDH (loading control) in the total protein lysates, generated using TBS with 1% Triton X-100, in the same variants of MC-38 and B16-F10 cells treated (+) or not (-) with IFNγ. **(I)** Muring TAPBPR can still associate with calnexin, albeit weakly, in the absence of association with MHC-I. The lanes highlighted in the red boxes represent independent repeats of the endogenous TAPBPR immunoprecipitations in WT and β2m-deficient MC-38 cells shown in Figure 2E. **(J)** Immunoprecipitation with H2-K^b and H2-D^b specific antibodies confirm both allotypes interact well with β2m. Immunoblots indicating abundance of MHC-I, H-2K^b, H-2D^b, β2m, tapasin, and GAPDH (loading control) in the whole cell lysate and the H-2K^b or H-2D^b immunoprecipitates (IP) on variants of MC-38 cells. Antibody only indicates the detection of antibody heavy chain of the antibody used in the immunoprecipitation. A,H&J are representative of three independent repeats.

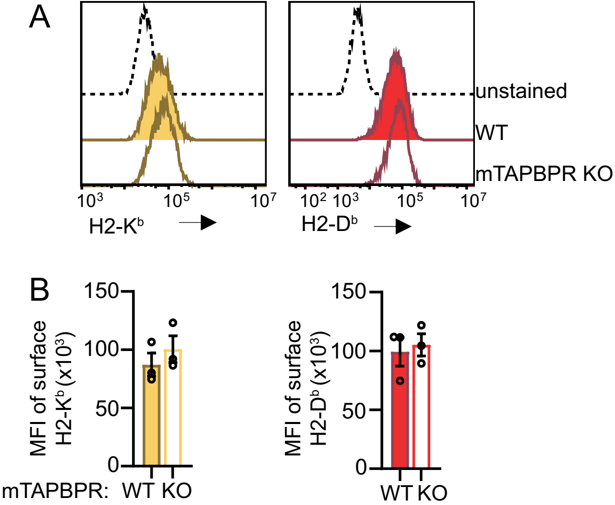

Supplementary Figure 3: Depletion of TAPBPR expression in MEF cells does not affect H2-D^b or -K^b expression. (A) Representative histograms and **(B)** bar charts graphs showing mean fluorescence intensity (MFI) of cell surface expressed H2-K^b or H2-D^b in WT and TAPBPR knockdown cells treated with IFNγ. Error bars show MFI -/+ SEM. Results from three independent experiments are shown.

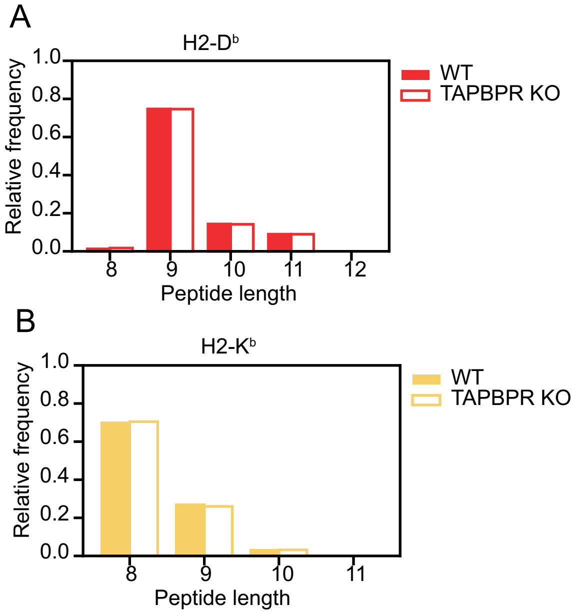

Supplementary Figure 4: Depletion of TAPBPR does not affect peptide length presented on H2-D^{b} or H2-K^{b} in MC-38 cells. Bar graphs showing the frequency of different lengths of peptides unique to WT and TAPBPR knockout cells presented on (**A**) H2-D^{b} and (**B**) H2-K^{b}.

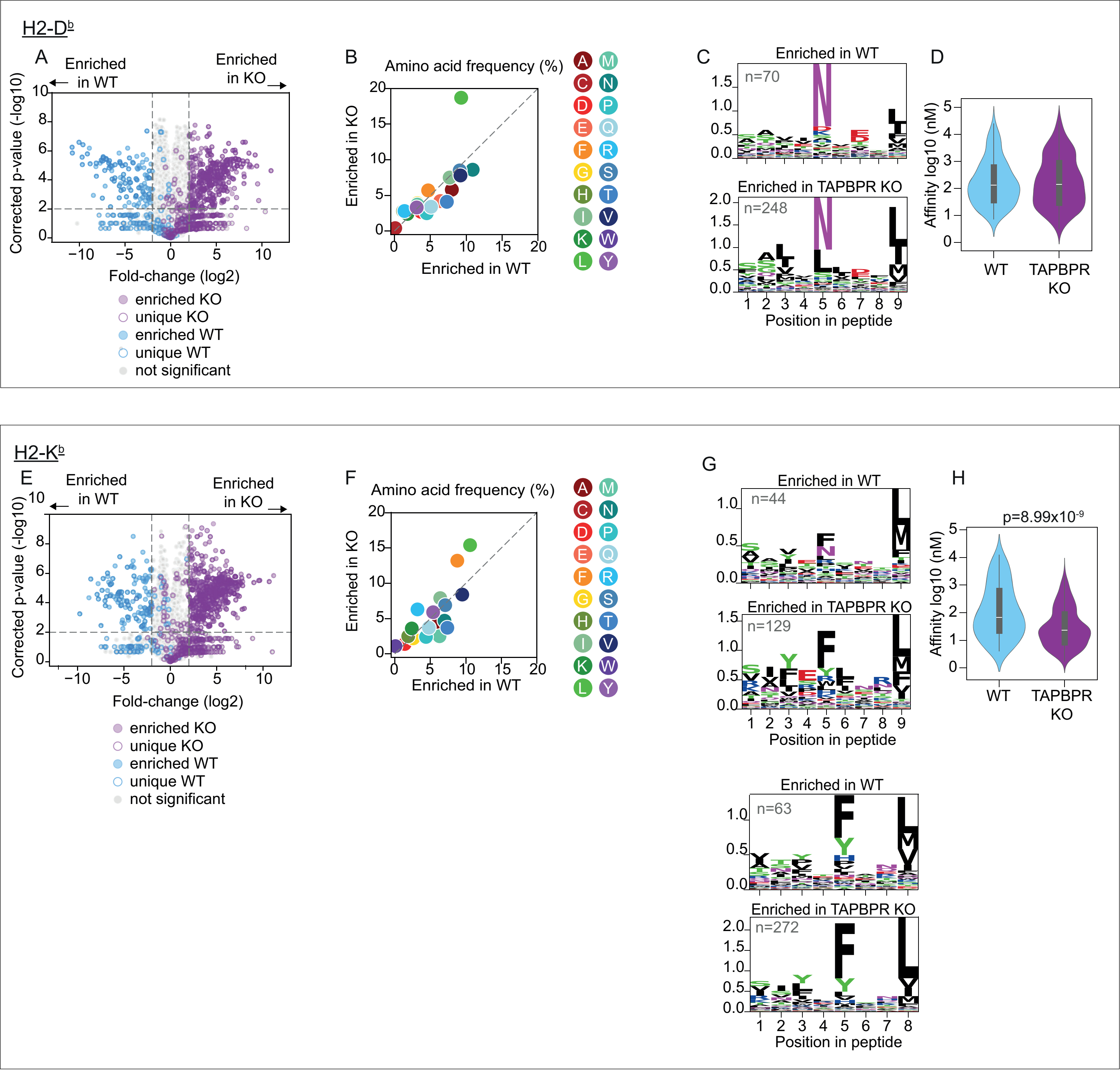

Supplementary Figure 5: MC-38 immunoepeptidomics analysis on enriched peptides. Analysis of peptides enriched in IFNγ treated WT and TAPBPR knockout (KO) MC-38 cells expressing (**A-D**) H2-D^b and (**E-H**) H2-K^b. (**A,E**) Volcano plots showing peptides enriched in WT and TAPBPR knockout cells. Blue and purple dots indicate peptides statistically significantly enriched in WT and TAPBPR knockout cells, respectively. (**B,F**) Frequency (%) of each amino acids presented on peptides enriched on WT or TAPBPR knockout on H2-D^b and H2-K^b. (**C, F**) SeqLogos showing the consensus sequences of 9mer (both H2-D^b and -K^b) ND 8mer (H2-K^b only) peptides enriched on WT and TAPBPR KO cells. (**D,H**) Violin plots show the NetMHC-predicted affinities of the peptides enriched to TAPBPR WT and TAPBPR KO cells for H2-D^b and H2-K^b. Statistics obtained using a Mann-Whitney U test.

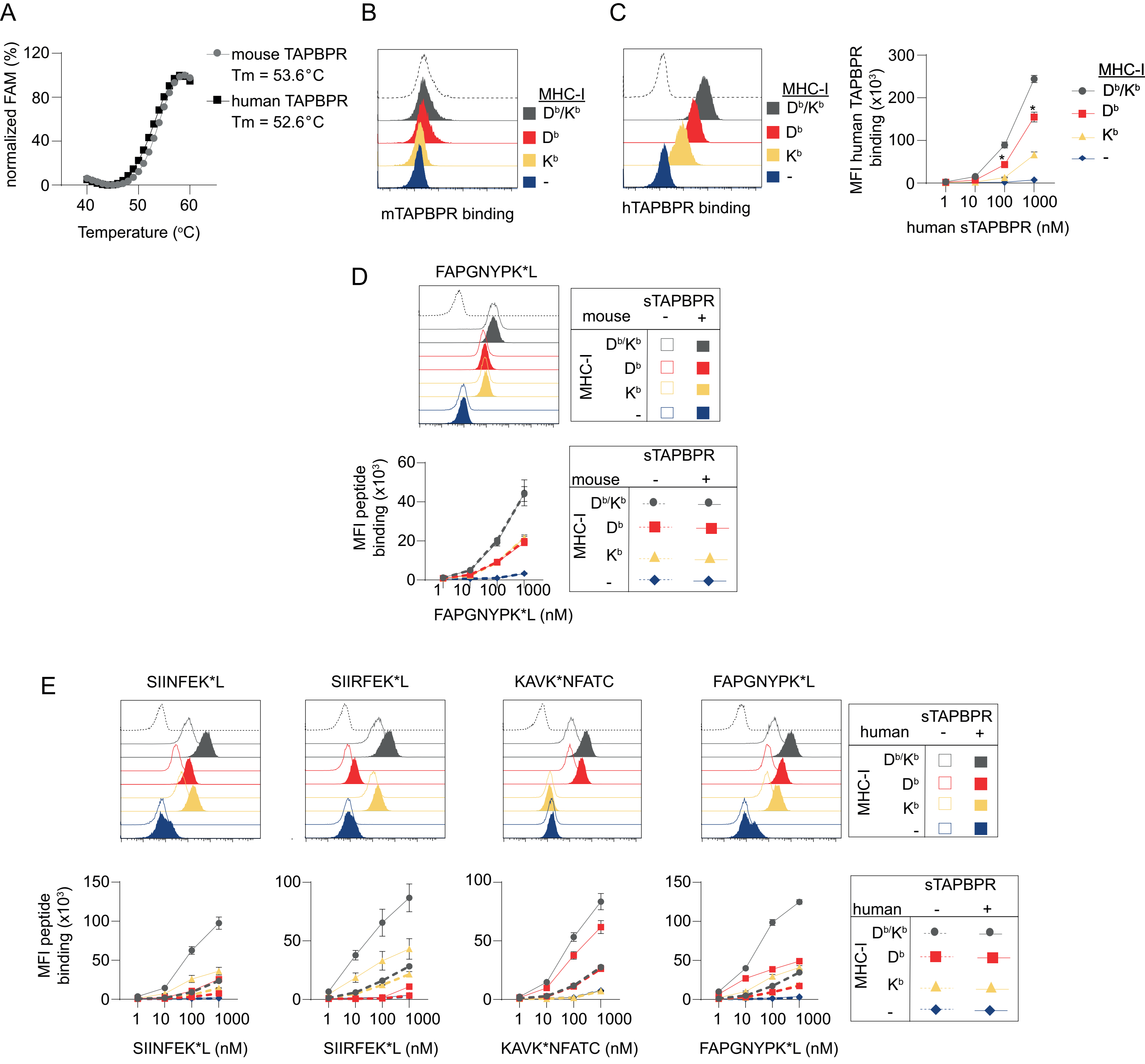

Supplementary Figure 6: Soluble mouse TAPBPR cannot be used to test peptide exchange on H2-D^b and K^b. (**A**) Differential scanning fluorimetry showing the melting temperature of recombinant mouse TAPBPR (grey) compared to recombinant human TAPBPR (black). (**B,C**) Recombinant mouse TAPBPR does not bind to MHC-I expressed on MC-38 cells, whereas recombinant human TAPBPR does. Representative histogram show binding of recombinant (**B**) mouse and (**C**) human TAPBPR to the surface of IFNγ-treated MC-38 cells incubated with 100 nM of each recombinant proteins. The line graph in C depicts the Mean fluorescence intensity (MFI) of human TAPBPR binding to MC-38 cell lines treated with 1-1000 nM of recombinant protein. Binding is shown for WT MC-38 cells expressing both H2-D^b and H2-K^b (grey), as well as for variant lines expressing H2-D^b only (red), H2-K^b only (yellow) and lacking β2m, and consequently deficient in surface H2-D^b and -K^b expression (blue). Staining with secondary antibody alone is shown as a dashed line. (**D,E**) Recombinant mouse TAPBPR does not load peptide onto MHC-I on MC-38 cells, while human TAPBPR does. Representative histogram and line graphs show MFI of peptide binding to IFNγ-treated WT MC-38 cells (grey), or variants expressing H2-D^b only (red), H2-K^b only (yellow) and β2m-deficient (blue). Cells were treated with or without 1000 nM of (**D**) mouse or (**E**) human soluble TAPBPR, followed by incubation with 1-1000 nM of FAPGNYPK*L, SIINFEK*L, SIIRFEK*L, or KAVK*NFATC, as indicated. Histograms include cells not treated with peptide (dotted line) as a control to represent baseline fluorescence. Error bars represent SEM. Data are presentative of three independent experiments performed in duplicate. Statistics obtained using ordinary one-way ANOVA with Tukey's multiple comparison.

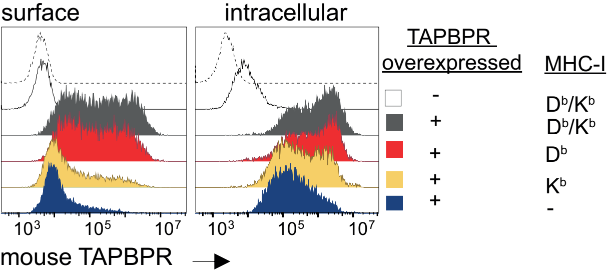

Supplementary Figure 7: Overexpressed mouse TAPBPR leaks to the plasma membrane in MEF cells. Representative histograms of surface and intracellular TAPBPR on MEF-BL/6-1 cells transduced with mouse TAPBPR. Mouse TAPBPR was overexpressed in WT cells (grey), cells expressing H-2D^{b} only (red), cells expressing H-2K^{b} only (yellow) or β2m knockdown cells (blue). TAPBPR levels on WT MEF-BL/6-1 (unfilled, grey histrogram) and unstained cells are included as controls (dashed line).
